# The Role of Secreted Frizzled-Related Protein 5 (Sfrp5) in Overweight and Obesity in Childhood and Adolescence

**DOI:** 10.3390/nu16183133

**Published:** 2024-09-17

**Authors:** Diamanto Koutaki, George Paltoglou, Maria Manou, Aikaterini Vourdoumpa, Eleni Ramouzi, Anastasia-Maria Tzounakou, Athanasios Michos, Flora Bacopoulou, Emilia Mantzou, Emmanouil Zoumakis, Marina Papadopoulou, Penio Kassari, Evangelia Charmandari

**Affiliations:** 1 Division of Endocrinology, Metabolism and Diabetes, First Department of Pediatrics, National and Kapodistrian University of Athens Medical School, “Aghia Sophia” Children’s Hospital, 11527 Athens, Greece; mk_madw@hotmail.com (D.K.); gpaltoglou@gmail.com (G.P.); mariamanou93@hotmail.com (M.M.); katvourdouba@gmail.com (A.V.); eleni_ramouzi@hotmail.gr (E.R.); anastasia.tzounakou@gmail.com (A.-M.T.); amantzou@med.uoa.gr (E.M.); mzoumak@med.uoa.gr (E.Z.); marinageorpap@gmail.com (M.P.); peniokassari@gmail.com (P.K.); 2 Department of Hygiene and Epidemiology, School of Medicine, University of Ioannina, 45110 Ioannina, Greece; 3 Division of Infectious Diseases, First Department of Pediatrics, National and Kapodistrian University of Athens Medical School, “Aghia Sophia” Children’s Hospital, 11527 Athens, Greece; amichos@med.uoa.gr; 4 University Research Institute of Maternal and Child Health and Precision Medicine, and UNESCO Chair on Adolescent Health Care, National and Kapodistrian University of Athens, “Aghia Sophia” Children’s Hospital, 11527 Athens, Greece; bacopouf@hotmail.com; 5 Division of Endocrinology and Metabolism, Center of Clinical, Experimental Surgery and Translational Research, Biomedical Research Foundation of the Academy of Athens, 11527 Athens, Greece

**Keywords:** obesity, overweight, Sfrp5, childhood, adolescence

## Abstract

**Background/Objective:** Secreted frizzled-related protein 5 (Sfrp5) is an anti-inflammatory adipokine that has been implicated in the pathophysiology of obesity and its metabolic complications. Despite the fact that numerous studies have been carried out in adults, limited data on Sfrp5 exist for youth, especially in relation to overweight and obesity. **Methods:** In our study, we assessed the concentrations of Sfrp5, total oxidative (TOS) and antioxidative (TAS) status, high-sensitivity *C*-reactive protein (hs-CRP), and several cytokines (IL-1α, IL-1β, IL-2, IL-6, IL-8, IL-12, TNF-α) in 120 children and adolescents (mean age ± SE: 11.48 ± 0.25 years; 48 prepubertal, 72 pubertal; 74 males and 46 females) before and 1 year after the implementation of a personalized, structured, lifestyle intervention program of healthy diet, sleep, and physical exercise. **Results:** Based on the body mass index (BMI), participants were categorized as having morbid obesity (*n* = 63, 52.5%), obesity (*n* = 21, 17.5%), overweight (*n* = 22, 18.33%), or normal BMIs (*n* = 14, 11.67%), based on the International Obesity Task Force (IOTF) cut-off points. Following the 1-year lifestyle intervention program, a significant improvement in anthropometric measurements (BMI, BMI-z score, diastolic blood pressure, WHR, and WHtR), body-composition parameters, hepatic enzymes, lipid profile, inflammation markers, and the insulin-sensitivity profile (HbA1C, HOMA index) was observed in all subjects. Sfrp5 decreased in subjects with obesity (*p* < 0.01); however, it increased significantly (*p* < 0.05) in patients with morbid obesity. Linear regression analysis indicates that TNF-α and systolic blood pressure were the best positive predictors and hs-CRP was the best negative predictor for Sfpr5 concentration at initial assessment and glucose concentration for ΔSfrp5, while TNF-α and TAS were the best positive predictors for Sfpr5 concentration at annual assessment. **Conclusions:** These results indicate that Sfrp5 is associated with severe obesity and is increased following weight loss in children and adolescents with morbid obesity. It is also related to metabolic homeostasis, as well as inflammation and oxidative status.

## 1. Introduction

Obesity has become a significant health problem of our time, and it is rapidly evolving into a pandemic. The prevalence of obesity worldwide more than doubled from 1990 to 2022 [1]. According to the International Classification of Diseases (ICD), obesity is defined as a chronic, relapsing, multifactorial disease [2]. Excessive fat accumulation in human tissues is a marker of it, which in turn leads to a multitude of negative effects on human health. The swift rise in childhood obesity is of particular concern, given that it not only poses immediate health risks but also sets the stage for long-term consequences.

In addition to being responsible for energy conversion and storage, adipose tissue represents a dynamic endocrine organ [3]. Given its dimensions, it is the body’s biggest endocrine gland, producing hormones referred to as adipokines. Adipokines are intermediaries with endocrine, paracrine, and autocrine actions, as well as pro-inflammatory or anti-inflammatory actions. More than 600 adipokines have been described, including leptin, adiponectin, visfatin, ghrelin, and resistin [4]. The positive energy balance observed in obesity leads to a substantial malfunction of adipose tissue. Adipocytes multiply (hyperplasia) and become larger (hypertrophy), remodeling of the stroma is observed, and the adipokine secretion profile is altered, resulting in low-grade chronic inflammation [5,6]. This prolonged inflammation, in turn, triggers a host of complications, such as dyslipidemia, endothelial dysfunction, hypertension, insulin resistance, diabetes mellitus type 2, fatty-liver disease, and malignancies [7]. Adipokines are important players in the etiology of obesity and its related disorders by regulating the equilibrium between anti-inflammatory and pro-inflammatory actions and acting on nearly all of the human body’s systems. Therefore, the investigation of adipokines and the mechanism by which they act is of particular interest. Adipokines enable us not only to better understand the underlying pathogenetic mechanisms of these complications but also to investigate novel treatments.

A recently recognized adipokine, first described by Ouchi et al., is secreted frizzled-related protein 5 (Sfrp5) [8]. Sfrp5 is a member of the Sfrp proteins, which in humans include five members produced in a variety of tissues, such as the subcutaneous, visceral, and pericardial adipose tissue, the heart, the liver, the pancreas, and mononuclear blood cells [9]. Sfrp5 is secreted by healthy adipocytes; it serves as an essential mediator in the Wnt (wingless-related integration site) signaling pathway and preserves anti-inflammatory and insulin-sensitizing properties. It possesses an *N*-terminal cysteine-rich domain (CRD), which shares a high degree of homology to wingless-type (Wnt) receptor frizzled proteins, and a small *C*-terminal hydrophilic domain [10]. Sfrp5 influences several cellular processes, such as differentiation, proliferation, and migration, by opposing extracellular Wnt proteins and preventing them from interacting with the Frizzled (Frz) receptor [11]. Furthermore, it promotes adipogenesis and serves as an indicator of mature adipocytes [12].

Low-grade, chronic inflammation is linked to obesity, and adipokine dysfunction has a significant impact on its promotion. Therefore, it is speculated that Sfrp5 possibly contributes to the pathophysiology of obesity and its complications. Research has been performed elucidating Sfrp5’s role in obesity. Animal studies have indicated a decrease in the expression of Sfrp5 in obese models, while Sfrp5 deficiency causes insulin resistance, glucose intolerance, and hepatosteatosis [8,13]. Clinical studies in adults suggest that Sfrp5 plays a prominent role in regulating adipogenesis, inflammation, glucose homeostasis, lipid metabolism, cardiovascular disease, non-alcoholic fatty-liver disease (NAFLD), and oxidative stress [14,15,16,17]. However, limited data exist concerning the association of Sfrp5 with obesity and its comorbidities in children and adolescents [18,19,20,21].

Although sufficient evidence suggests that there is downregulation of Sfrp5 in obesity and upregulation after weight loss, much research has revealed contradicting findings [12,14,22,23,24]. Therefore, deeper research studies are needed to clarify how excess fat regulates Sfrp5 and how it is affected along with alterations in body mass index (BMI), especially in children. Studies in children and adolescents offer us the opportunity to improve our insight into the pathogenesis of obesity in the initial periods of its development without the confounding effects of medications and/or metabolic complications.

The aim of our prospective study was to investigate the effect of a 1-year personalized, comprehensive, structured, multidisciplinary, lifestyle-intervention program of diet, sleep, and exercise on Sfrp5 concentrations in children and adolescents with obesity, overweight, and normal BMIs. Furthermore, we aimed to address potential associations of Sfrp5 with cardiovascular risk factors and glucose metabolism.

## 2. Patients and Methods

### 2.1. Study Population and Ethics

The present study was prospectively designed. The study population consisted of one hundred and twenty (*n* = 120; 48 prepubertal, 72 pubertal; 74 males and 46 females) children and adolescents who were recruited from our Center for the Prevention and Management of Overweight and Obesity in Childhood and Adolescence, Division of Endocrinology, Metabolism, and Diabetes, First Department of Pediatrics, National and Kapodistrian University of Athens Medical School. The study was conducted in accordance with the Declaration of Helsinki. The study was approved by the ethics committee of ‘Aghia Sophia’ Children’s Hospital (Approval Number: EB-PASCH-MoM: 7 April 2021; Re:10997/26/02/2021). All parents and guardians were asked to sign an informed consent form after a full explanation of the study, and all children older than seven years old provided their assent. Based on BMI, subjects were classified as having morbid obesity (*n* = 63, 52.5%), obesity (*n* = 21, 17.5%), overweight (*n* = 22, 18.33%), or normal BMIs (*n* = 14, 11.67%), based on the International Obesity Task Force (IOTF) cut-off points [25]. Exclusion criteria were (i) chronic or acute diseases, (ii) endocrine disorders, (iii) obesity due to a known monogenic cause or in the context of a syndrome, (iv) received corticosteroids, antiepileptics, or other preparations that increase BMI, and (v) malnutrition [BMI Standard Deviation Score (SDS) ≤ −2].

All participants were assessed at frequent intervals by a multidisciplinary team that included a Pediatrician, a Pediatric Endocrinologist, a Pediatric Dietician, a professional fitness Personal Trainer, and—when necessary—a Pediatric Clinical Psychologist. All subjects joined an individualized management program, which offered guidance and assistance to both participants and their families regarding healthy dietary choices, sleep, and exercise [26,27,28,29,30,31].

### 2.2. Methods

#### 2.2.1. Anthropometric and Body-Composition Parameters

Body weight was measured wearing light clothes and without shoes with the same calibrated scale (Seca GmbH & Co. KG., Hamburg, Germany). Standing height was also measured without shoes with a stadiometer (Holtain Limited, Crymych–Dyfed, UK). Weight and height were used to calculate body mass index as body weight (kg)/height (m^2^). Consequently, the BMI *z*-score was calculated according to the Greek standard growth charts [32]. Furthermore, the WHO STEPS protocol was used for waist and hip circumference measurements by employing the same stretch-resistant tape in standing position (Seca GmbH & Co. KG., Hamburg, Germany) in the horizontal plane midway between the lowest rib and the iliac crest at the end of a normal expiration, and in the horizontal plane at the level of maximum circumference of hips and buttocks, respectively. Resting blood pressure (BP) measurement was assessed by a sphygmomanometer with an appropriate cuff for arm circumference (Comfort 20/40, Visomat, Parapharm, Metamorphosi, Attiki, Greece). Additionally, bioelectrical impedance analysis (BIA) was performed on each individual. (TANITA MC-780U Multi Frequency Segmental Body Composition Analyzer, Amsterdam, The Netherlands).

#### 2.2.2. Initial Assessment and Interventions

On the day of the study, all subjects were admitted to the Endocrine Unit early in the morning, and a comprehensive medical history and clinical examination were performed, including evaluation of pubertal status; standard anthropometric measurements (weight, height, WC, and HC) were gathered by a single trained observer. At 08:00 a.m., blood samples for initial hematologic, biochemical, and endocrinologic examinations, and Sfrp5 concentrations were collected, following an overnight 12 h fast. Then, they were centrifuged, immediately separated after collection, and stored at −80 °C until assayed. Furthermore, body-composition analysis was performed.

Subsequently, all participants were evaluated by a Pediatrician and a Pediatric Dietitian regarding their daily nutritional habits. Using the United States Department of Agriculture (USDA) method, a 24 h recall of their meals was completed, detailing food and beverage intake, as well as their quantity and timing [33]. This information was used to assess dietetic habits and nutritional status. Children and their parents/guardians received detailed information on the implications of obesity and the significance of adopting a healthier lifestyle as a family unit. Guidance was provided on modifying dietary habits, emphasizing the reduction of processed food consumption and the incorporation of fresh fruits, vegetables, and whole grains in accordance with the 2010 USDA guidelines and recommendations from the National Nutritional Guide for Infants, Children, and Adolescents [27,28,29,33]. In addition, detailed recommendations for adequate sleep were provided to each participant based on their age, according to the American Academy Consensus Guidelines [34]. These guidelines suggested 9 to 12 h of sleep per day for children aged 10–12 years and 8 to 10 h per day for adolescents aged 13–18 years. Participants were advised to prioritize uninterrupted sleep, aiming to start sleeping as early as possible before midnight and maintaining a consistent sleep schedule every day. Also, children were advised to limit screen time to less than two hours per day and to turn off all electronic devices one hour before bedtime.

Additionally, a certified Personal Trainer assessed each participant on their initial visit, and they recorded their weekly activities and interests. His role included designing and implementing tailored exercise programs for the children, providing guidance on physical activities and sports, motivating the children to engage in regular exercise, and promoting the importance of leading an active lifestyle [27,28,29].

Subjects with obesity were evaluated every month, those overweight every two months, and those with normal BMIs every three months. During each follow-up visit, anthropometric measurements were acquired, and a new 24 h diet recall was conducted, as previously described. All health professionals discussed the progress, adjusted goals, and encouraged the active involvement of parents and guardians in the process to provide additional support to children and adolescents. Detailed hematologic, biochemical and endocrinologic tests were carried out at the beginning and the end of the study, as well as at 3–6 monthly intervals as required [26,27,28,29,30,31].

#### 2.2.3. Annual Assessment

On the day of the annual follow-up visit, all participants were admitted to the Endocrine Unit early in the morning. A single trained observer collected standard anthropometric metrics (weight, height, WC, and HC), as well as an extensive medical record and physical exam, which included a Tanner pubertal examination. After a fasting period of twelve hours, thorough hematologic, biochemical, and endocrinologic tests were conducted at 8:00 a.m., along with a body-composition study after the clinical evaluation.

#### 2.2.4. Assays

The ADVIA 2110i analyzer was used for standard hematologic investigations (Roche Diagnostics GmbH, Mannheim, Germany). The concentrations of total cholesterol, high-density lipoprotein cholesterol (HDL), triglycerides (TG), and glucose were measured in the analyzer ADVIA 1800 Siemens (Siemens Healthcare Diagnostics, Tarrytown, NY, USA), while apolipoproteins A1 (ApoA1), B (ApoB) and lipoprotein (a) [Lp(a)] concentrations were measured by means of latex particle-enhanced immunonephelometric assays on the BN ProSpec nephelometer (Dade Behring, Siemens Healthcare Diagnostics, Liederbach, Germany). Hemoglobin A1C (HbA1C) was measured with the employment of reversed-phase cation exchange high-performance liquid chromatography on an HA-8160 automated glycohemoglobin analyzer (Arkray, Kyoto, Japan).

To estimate insulin resistance, the homeostasis model assessment (HOMA-IR) method was calculated as follows: HOMA-IR = [fasting glucose (mg/dL) × fasting insulin (mU/L)]/405. The 2009 bedside Schwartz formula was applied for the calculation of the estimated glomerular filtration rate (eGFR) as follows: eGFR = 0.413 × height (cm)/creatinine (mg/dL).

The concentrations of luteinizing hormone (LH), follicle-stimulating hormone (FSH), ferritin, estradiol, and insulin were determined using an automated electrochemiluminescense immunoassays analyzer (Cobas e411, Roche Diagnostics GmbH, Mannheim, Germany), while thyroid-stimulating hormone (TSH), free thyroxine, anti-thyroid peroxidase antibodies, anti-thyroglobulin antibodies, adrenocorticotropin (ACTH), cortisol, testosterone, androstenedione, dehydroepiandrosterone (DHEA), dehydroepiandrosterone sulfate (DHEAS), insulin-like growth factor-I (IGF-I), and high-sensitivity *C*-reactive protein (hs-CRP) concentrations were determined using chemiluminescence immunoassays on an IMMULITE 2000 immunoassay system (Siemens Healthcare Diagnostics Products Ltd., Camberley, Surrey, GU15 3YL, K). The total 25-hydroxyvitamin D (25-OH-Vitamin D) concentration was measured by an automated electrochemiluminescence immunoassay with the Modular Analytics E170 analyzer (Roche Hellas, Athens, Greece).

The concentrations of Sfrp5 were determined by ELISA Enzyme-Linked Immunosorbent Assay (SEC842Hu, Cloud-Clone Corp., Katy, TX 77494, USA) with sensitivity limits of 0.58 ng/mL. The total oxidative status (TOS) and total antioxidant status (TAS) were determined using a photometric test system [PerOx(TOS) kit KC510, Immundiagnostik AG, ImAnOx(TAS) kit KC5200, Immundiagnostik AG, Stubenwald-Allee 8a, 64625 Bensheim, Germany]. The cytokines IL-1α, IL-1β, IL-2, IL-6, IL-8, IL-12, and tumor-necrosis factor-α (TNF-α) were determined by ELISA Enzyme-Linked Immunosorbent Assay [DLA50, HSLB00D, HS200, HS600C, HS800, HS120, HSTA00E respectively, Quantikine ELISA, R&D Systems (Minneapolis, MN, USA)].

#### 2.2.5. Statistical Analysis

The statistical analysis was carried out utilizing Statistica 14 software [TIBCO Software Inc. (2020), Palo Alto, CA, USA]. All variables were normally distributed. Results are expressed as mean ± standard error of the mean (SE). The statistical significance level was set at *p* < 0.05. At initial assessment, anthropometric parameters were compared among subjects with morbid obesity, obesity, overweight, and normal BMIs, with a one-factor analysis of variance test (one-factor ANOVA). The effects of lifestyle intervention for all variables assessed at the beginning of the study (‘initial assessment’) and after 1 year (‘annual assessment’) were compared by employing a repeated-measures analysis of variance test (repeated-measures ANOVA) with and without obesity/overweight/normal BMI as between subjects’ factors. Significant main effects were revealed by Fischer’s Least Significant Difference (LSD) post-hoc test. The associations of the studied variables were studied by Pearson’s R coefficient.

Standard forward stepwise multiple linear regression models were applied to examine possible predictors of Sfrp5, initially and annually (mean), and the respective change (delta) of Sfrp5 (ΔSfrp5), all taken separately as dependent variables. In the first model, inflammation parameters (TAS, TOS, hs-CRP, TNF-α, and IL-6) at initial assessment were used as independent variables. In the next model, the metabolic-syndrome parameters [Systolic Blood Pressure (SBP), WC, glucose concentration, TG, and High-Density Lipoprotein (HDL)] at initial assessment were used as independent variables. In the last model, the metabolic syndrome parameters (WC, SBP, glucose concentration, TG, and HDL) at initial assessment were used as independent variables for ΔSfrp5.

## 3. Results

### 3.1. Clinical Characteristics and Body Composition, Biochemical, and Endocrinologic Parameters

A total of 120 [48 prepubertal (40%) and 72 pubertal (60%); 74 males (62%) and 46 females (38%)] children and adolescents aged 6–18 years old (mean age ± SE: 11.48 ± 0.25 years) were studied prospectively. The clinical characteristics of all participants were categorized as follows: having morbid obesity, obesity, overweight, or a normal BMI based on IOTF criteria at baseline and are presented in Table 1. The clinical characteristics after 6 months and 1 year of the lifestyle intervention program was implemented are presented in Table 2. More specifically, body weight (*p* < 0.01), BMI (*p* < 0.01), BMI-z score (*p* < 0.01), diastolic blood pressure (DBP) (*p* < 0.01), WC (*p* < 0.01), HC (*p* < 0.01), waist-to-hip ratio (WHR) (*p* < 0.05) and waist-to-height ratio (WHtR) (*p* < 0.01) decreased significantly at the 6-month and annual assessments. Furthermore, BMI *z*-score (*p* < 0.01), DBP (*p* < 0.01), and WHtR (*p* < 0.01) also decreased significantly from the 6-month to the 12-month assessment.

Biochemical, endocrinologic, and body-composition parameters at initial and annual assessment are presented in Table 3. After the implementation of the lifestyle intervention program, all participants showed a significant decrease in the concentrations of glucose (*p* < 0.01), hepatic enzymes [(aspartate aminotransferase (AST) (*p* < 0.05), alanine transaminase (ALT) (*p* < 0.01), gamma-glutamyl transferase (γGT) (*p* < 0.01)], triglycerides (*p* < 0.05), and ApoB (*p* < 0.01), and a significant increase in the concentrations of HDL (*p* < 0.01), Lp(a) (*p* < 0.01), and 25-OH-Vitamin D (*p* < 0.01). Indicators of insulin sensitivity, including fasting serum insulin concentrations, HbA1C, and HOMA-IR (*p* < 0.01), improved significantly after the implementation of the multidisciplinary lifestyle intervention program. When analyzing the body composition of all patients, fat percentage and fat mass decreased significantly (*p* < 0.01), whereas fat free mass, muscle mass percentage, bone mass, total body water, and basal metabolic rate (BMR) increased (*p* < 0.01) at the annual assessment.

### 3.2. Adipose Tissue Inflammation-Associated Factors and Subgroups Analysis

**Figure 1:**
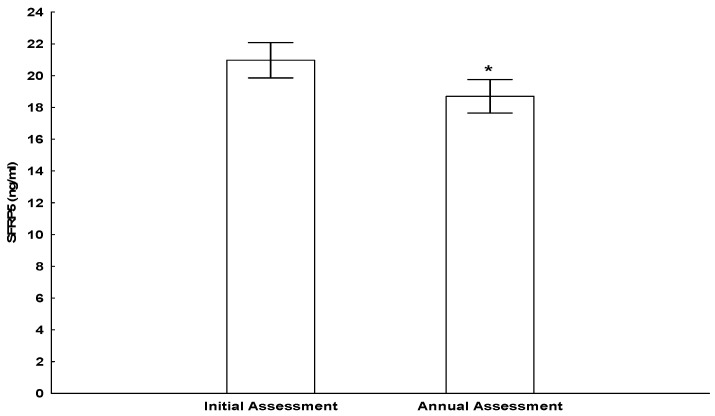
Concentrations of Sfrp5 at initial and annual assessment. The asterisk (*) indicates statistically significant difference.

**Figure 2:**
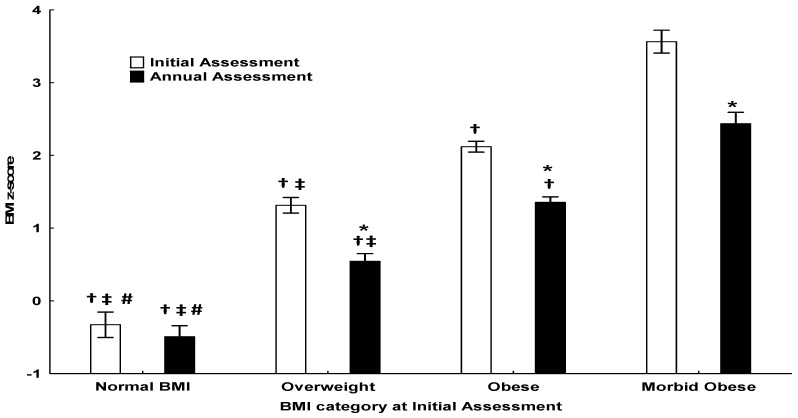
BMI *z*-score at initial and annual assessment based on BMI category. *: indicates significant difference from initial assessment. †: indicates significant difference from subjects with morbid obesity. ‡: indicates significant difference from subjects with obesity. #: Indicates significant difference from subjects with overweight.

The concentrations of adipokine Sfrp5 (Figure 1), as well as interleukins and hs-CRP at initial and annual assessments, are presented in Table 4. There was a significant decrease in hs-CRP (*p* < 0.05), Sfrp5 (*p* < 0.01), and TOS (*p* < 0.01), and a significant increase in TNF-α (*p* < 0.01), IL-1β (*p* < 0.01), and IL-8 (*p* < 0.01) concentrations after the lifestyle intervention program was implemented for 1 year. The comparisons of clinical, biochemical, and endocrinologic parameters, adipose tissue inflammation-associated parameters, and body-composition parameters according to BMI are presented in Table 5. BMI *z*-score at each subgroup initially and annually is also presented in Figure 2. There was no significant difference in Sfrp5 concentrations among the groups; however, Sfrp5 increased in subjects with morbid obesity following weight loss (*p* < 0.05). TOS was significantly higher in the morbid obesity group compared to the normal BMI group, both at initial and annual assessment, and it decreased significantly after weight loss (*p* < 0.05).

SBP and DBP were higher in subjects with morbid obesity and decreased significantly following weight loss (*p* < 0.05). The concentrations of ALT (*p* < 0.05) and γGT (*p* < 0.05) decreased significantly, while AST decreased in patients with obesity (*p* < 0.05). HDL increased (*p* < 0.05), and ApoB (*p* < 0.05) decreased in the morbid obesity and obesity subgroups, while TG decreased in the morbid obesity group (*p* < 0.05). Insulin sensitivity was lower in the morbid obesity/obesity group in comparison with the normal-BMI group initially and annually, as evidenced by insulin, HbA1C, and HOMA-IR levels. Furthermore, a significant improvement was noted in these indices in the morbid obesity/obesity group at the 12-month follow-up.

### 3.3. Correlation Coefficient Analysis According to BMI

The correlation coefficient analysis for Sfrp5 is presented in Supplemental Table S1. In the normal-BMI group, Sfrp5 concentrations correlated positively with TG (r = 0.665, *p <* 0.05), insulin (r = 0.611, *p <* 0.05), HOMA index (r = 0.553, *p <* 0.05), TNF-α (r = 0.699, *p <* 0.05) and IL-6 (r = 0.569, *p <* 0.05) concentrations, as well as fat mass (r = 0.614, *p <* 0.05), and negatively correlated with HDL (r = −0.623, *p <* 0.05) and ApoA1 (r = −0.565, *p <* 0.05) concentrations. In the overweight group, Sfrp5 concentrations correlated positively with IL-8 (r = 0.488, *p <* 0.05) concentrations. In the obesity group, Sfrp5 concentrations correlated positively with BMI (r = 0.48 *p <* 0.05), SBP (r = 0.61, *p <* 0.05), and uric acid concentrations (r = 0.707, *p <* 0.05). In the morbid obesity group, Sfrp5 correlated positively with IL-12 concentrations (r = 0.604, *p <* 0.05). No significant correlation was observed between ΔSfrp5 and ΔBMI (*p >* 0.05).

### 3.4. Predictors of Sfrp5 and ΔSfrp5

When inflammation parameters at initial assessment (TAS, TOS, hs-CRP, TNF-α, and IL-6) in a standard forward stepwise regression model were used as independent variables, TNF-α concentration (b = 0.288, *p* < 0.05) was the best positive predictor, while hs-CRP was the best negative predictor (b = −0.232, *p* < 0.05) of Sfrp5 concentration. When inflammation parameters at the annual assessment (TAS, TOS, hs-CRP, TNF-α, and IL-6) were used in a standard forward stepwise regression model as independent variables, TNF-α concentration (b = 0.277, *p* < 0.05) was the best positive predictor of Sfrp5 concentrations, followed by TAS concentrations (b = 0.228, *p* < 0.05).

When metabolic syndrome parameters at initial assessment (glucose concentration, SBP, WC, TG, and HDL) were used as independent variables in a standard forward stepwise regression model, SBP (b = 0.233, *p* < 0.05) was the best positive predictor of Sfrp5 concentrations. When metabolic syndrome parameters at initial assessment (glucose concentration, SBP, WC, TG, and HDL) were used in a standard forward stepwise regression model as independent variables, glucose concentration (b = 0.208, *p* < 0.05) was the best positive predictor for the change of Sfrp5 (ΔSfrp5).

All the predictors are presented in Supplemental Table S2.

## 4. Discussion

Sfrp5 is a relatively novel adipokine with anti-inflammatory properties [35]. In our study, we examined the impact of a 1-year personalized, structured, comprehensive, multidisciplinary lifestyle intervention program of diet, sleep, and exercise in Sfrp5 concentrations in children and adolescents with morbid obesity, obesity, overweight, and normal BMIs. We demonstrated that Sfrp5 concentrations increased significantly in patients with morbid obesity after the lifestyle intervention program was implemented for 1 year. We also showed that TNF-α and SPB were the best positive predictors, and hs-CRP was the best negative predictor for Sfpr5 concentrations at the initial assessment, while TNF-α and TAS were the best positive predictors for Sfpr5 concentrations at the annual assessment, and glucose concentration was the best positive predictor for ΔSfrp5. In addition, there was a significant improvement in anthropometric measurements (decrease in BMI, BMI *z*-score, DBP, WHR, WHtR, and percentage of fat; increase in muscle mass and fat-free mass), hepatic enzymes (AST, ALT, and γ-GT), lipid profile (TG, ApoB; increase in HDL concentrations), inflammation markers (decrease in hs-CRP and TOS) and the insulin sensitivity profile (HbA1C and HOMA index) in all patients. To the best of our knowledge, this is a novel study investigating Sfrp5 concentrations in a pediatric population and the first one with a 12-month duration to evaluate prospective changes in Sfrp5.

Sfrp5 concentrations increased significantly following weight loss in the subgroup of subjects with morbid obesity, consistent with the presence of low-grade inflammation in these patients [36]. This upregulation of Sfrp5 is consistent with prior research that examined the impact of an intervention program in children [19,20] and adults [34,35] with obesity. The reverse has been shown in animal models, where a high-fat/high-sucrose diet for 24 weeks resulted in decreased expression of Sfrp5 in leptin-deficient (ob/ob) mice and wild-type (WT) mice [8]. These findings suggest that Sfrp5 may be able to reverse the effect of obesity, owing to its ability to sequester WNT5A [wingless-type mouse mammary tumor virus (MMTV) integration site family member 5A] from the Frz receptor and mitigate the inflammation caused by WNT5A. This difference was noted only in the morbid obesity group and can be attributed to the fact that these patients have a greater degree of inflammation, as indicated by the higher concentrations of IL-6 in our study, especially in children who are relatively free of the complications of obesity. This elevation of Sfrp5, which was evident only in the morbid obesity subgroup, is consistent with prior research in children with obesity (defined as exceeding the 90th and 95th percentile) [19,20]. It is likely that the selection of the study population in previous studies (mainly children with obesity), could explain why the increased Sfrp5 concentrations were noted only in the morbid obesity subgroup. In our study we applied Greek standards based on the proposal of IOTF. Furthermore, the increase in Sfrp5 concentrations in patients with morbid obesity was accompanied by improvement in clinical parameters (decreased BMI, BMI z-score, DBP, WHR, WHtR, and percentage of fat percentage; increased muscle mass and fat-free mass), hepatic enzymes (decreased ALT, γ-GT), lipid profile (decreased TG, ApoB; increased HDL concentrations), and insulin-sensitivity indices (HbA1C, HOMA index).

According to our findings, Sfrp5 concentrations did not differ significantly between lean and obese subjects. It should be noted that concentrations of Sfrp5 in our study are lower compared to adult studies, but this can be attributed to the age difference of our study population, as other studies in children’s populations have also shown [18,19,20]. Although some previous studies have indicated a regulation of Sfrp5 by BMI [15,18,37,38], our results are consistent with several other studies that supported no difference in Sfrp5 concentrations between obese and lean subjects in adult patients [12,15,22,23]. This may be partially explained by the fact that the effect on serum Sfrp5 concentrations may differ from the effect on its expression in adipose tissue. Several studies indicated that Sfrp5 represents a marker of mature adipocytes rather than preadipocytes [12,22,39], while others suggested that it is not actively secreted from human white adipose tissue (WAT) [24]. Furthermore, Sfrp5 expression levels in animal studies were dependent on the duration of obesity [8,40]. The adipose tissue of mice fed a high-fat diet for a brief period of time showed higher levels of Sfrp5; however, when mice were given a high-fat diet over an extended length of time and the volume of adipocytes increased to a plateau stage, the Sfrp5 gene expression was downregulated. Jura et al. also proposed that the Sfrp5 gene expression decreased when the adiposity reached a plateau [40]. Thus, the correlation of Sfrp5 with BMI could be less evident, considering its time-dependent nature, especially in children with milder adipose-tissue inflammation.

After the 1-year lifestyle intervention program was implemented, fasting glucose concentration at baseline was the best predictor for the change of Sfpr5. This finding is consistent with prior research that has underscored the importance of Sfrp5 in glucose metabolism [15,21]. Sfrp5-deficient mice fed a high-fat, high-sucrose diet had impaired glucose metabolism [8]. In addition, Sfrp5 has a protective role in maintaining beta cell function while promoting glucose-dependent insulin secretion [41]. In 3T3-L1 pre-adipocytes, treatment with antidiabetic drugs, including metformin and rosiglitazone, increases Sfrp5 mRNA expression and protein secretion [39]. Hyperglycemia decreases circulating Sfrp5 concentrations during oral glucose tolerance tests (OGTT) in healthy and insulin-sensitive individuals, suggesting a direct effect of hyperglycemia [42]. These results concur with ours, given that glycemia predicts the decrease of Sfrp5 concentrations observed in all patients.

Our finding that Sfrp5 concentration is associated with blood pressure is also in accordance with other studies in children [20] and adults [15] with obesity, where Sfrp5 was negatively associated with SBP and DBP. Specifically, in vitro studies have shown that Sfrp5 exerts vasodilating properties by modulating nitric oxide (NO) production via inhibiting the WNT5A/JNK pathway in endothelial cells [43]. In rat thoracic aorta, treatment with Sfrp5 reversed the WNT5A-induced impaired vasorelaxation in a dose-dependent manner through an endothelial NO synthase-dependent pathway [38]. Thus, Sfrp5 may regulate blood pressure through the WNT signaling pathway.

Thus far, our knowledge of the association of Sfrp5 with interleukins and oxidative status has been scarce; therefore, we aimed to investigate this association in our study. TNF-α and TAS were the best positive predictors for Sfpr5 concentrations at initial and annual assessments, respectively, while hs-CRP was the best negative predictor. A high WNT5A/SFRP5 ratio, either in plasma or in perivascular adipose tissue surrounding human arteries, is related to significantly higher O_2_ generation in these vessels [44]. Srfp5 is closely related to antioxidant status and is associated with markers of oxidative stress [14]. In human aortic smooth-muscle cells (VSMCs), Sfrp5 protects against oxidative stress-induced apoptosis by inhibiting the Wnt/β catenin signaling pathway [45,46].

Hs-CRP is a well-established indicator of subclinical inflammation, and its relation to obesity is well-documented. We demonstrated that hs-CRP is a negative predictor of Sfrp5 concentrations. Macrophage infiltration is thought to be an indicator of adipose tissue inflammation, given that macrophages surround necrotic adipocytes to create characteristic crown-like structures (CLCs) [47]. In line with our findings, fat-biopsy specimens of subjects with obesity with macrophage CLSs presented lower Sfrp5 expression in comparison with subjects with obesity who were negative for CLS [8]. Furthermore, serum Sfrp5 concentrations in subjects with coronary artery disease (CAD) and adults with obesity were inversely related to hs-CRP concentrations [15,23,48]. These data are in accordance with the anti-inflammatory effects of Sfrp5 and may indicate its role in subclinical inflammation. However, in our study, TNF-α positively predicted Sfrp5 concentrations; TNF-α is produced by macrophages and monocytes but it also rises in reaction to higher levels of exercise in lean subjects and females [49,50]. Thus, we can speculate that this result is influenced by the inclusion of normal-BMI children and the status of physical fitness.

Our study presents notable strengths. To the best of our knowledge, this is one of the first studies investigating Sfrp5 concentrations in a pediatric population and the first one with a 12-month duration to evaluate prospective changes in Sfrp5. Furthermore, the exclusion of children and adolescents with chronic or acute disease offered us the opportunity to gain insight into the role of Sfrp5 in the initial periods of the pathophysiology of obesity. A limitation of this prospective study is the lack of a randomized control group without intervention, which is owing to the fact that all patients were consecutive attendees at our Center for the Prevention and Management of Childhood Obesity. The assessment of intervention program compliance based on the reported medical history is another limiting factor.

## 5. Conclusions

We conclude that Sfrp5 is related to obesity and, specifically, to morbid obesity in childhood and adolescence. An individualized lifestyle intervention program consisting of a healthy diet, good quality sleep, and physical exercise for 1 year resulted in a significant increase in Sfrp5 concentrations in children and adolescents with severe obesity, along with a significant amelioration in parameters of metabolic syndrome, such as body composition and clinical indices of obesity, hepatic enzymes, markers of inflammation, indices of insulin resistance, and profile of lipids. These results may indicate that Sfrp5 mediates weight loss and improves cardiometabolic risk parameters by reducing pro-inflammatory cytokines and promoting antioxidant status. Additional research is required to outline the physiological processes underlying Sfrp5 regulation related to BMI and dietary modification in the pediatric population.

## Figures and Tables

**Table 1:** (**A**) Clinical characteristics of all participants at baseline. Participants were categorized as having morbid obesity, obesity, overweight, or a normal BMI based on IOTF criteria at initial assessment. (**B**) Pubertal status and gender of subjects in each BMI category at initial assessment and the respective percentages in parentheses.

(A)					
	Morbid Obesity (*n* = 63)	Obesity (*n* = 21)	Overweight (*n* = 22)	Normal BMI (*n* = 14)	*p*
Age (years)	11.56 ± 0.37	11.14 ± 0.53	12.02 ± 0.52	10.75 ± 0.70	NS
Height (cm)	153.25 ± 1.96	153.19 ± 2.75	149.49 ± 1.98	145.80 ± 4.45	NS
BW (kg)	75.55 ± 3.11	63.31 ± 3.09 ^†^	54.30 ± 2.16 ^†^	39.24 ± 3.49 ^†,‡,#^	<0.01
BMI (kg/m^2^)	31.22 ± 0.68	26.57 ± 0.44 ^†^	24.07 ± 0.40 ^†,‡^	17.95 ± 0.72 ^†,‡,#^	<0.01
BMI *z*-score	3.56 ± 0.16	2.12 ± 0.07 ^†^	1.31 ± 0.11 ^†,‡^	−0.33 ± 0.17 ^†,‡,#^	<0.01
Waist circumference (cm)	97.98 ± 1.89	91.76 ± 1.84 ^†^	81.79 ± 1.40 ^†,‡^	66.38 ± 2.79 ^†,‡,#^	<0.01
Hip circumference (cm)	107.16 ± 1.94	96.18 ± 1.59 ^†^	89.55 ± 1.76 ^†^	79.42 ± 3.44 ^†,‡,#^	<0.01
WHR	0.91 ± 0.01	0.95 ± 0.01 ^†^	0.92 ± 0.02 ^‡^	0.84 ± 0.02 ^†,‡,#^	<0.01
SBP (mmHg)	110.46 ± 1.52	107.49 ± 3.22	103.34 ± 2.54 ^†^	94.54 ± 3.09 ^†,‡,#^	<0.01
DBP (mmHg)	73.47 ± 0.97	68.88 ± 2.09 ^†^	64.91 ± 2.16 ^†^	59.92 ± 2.04 ^†,‡^	<0.01
(**B**)					
**Pubertal Status**					**Total (*n* = 120)**
Pre-pubertal	24 (38%)	12 (57%)	6 (27%)	6 (43%)	48 (40%)
Pubertal	39 (62%)	9 (43%)	16 (73%)	8 (57%)	72 (60%)
**Gender**					
Male	35 (56%)	16 (76%)	13 (59%)	10 (71%)	74 (62%)
Female	28 (44%)	5 (24%)	9 (41%)	4 (29%)	46 (38%)

Abbreviations: BMI, body mass index; BW, body weight; DBP, diastolic blood pressure; SBP, systolic blood pressure; WHR, waist-to-hip ratio; all variables are presented as mean ± SE of mean. Participants were categorized as obese and overweight based on IOTF criteria at initial assessment. One-way ANOVA was used to compare all measurable variables. LSD post-hoc test indicated significant main effects. Statistical significance *p* < 0.05 and strong significance (*p* < 0.01) are presented. NS: nonsignificant difference (*p* > 0.05). ^†^: indicates significant difference from morbidly obese. ^‡^: indicates significant difference from obese. ^#^: indicates significant difference from overweight.

**Table 2:** Clinical characteristics of all participants at initial, 6-month, and annual assessments.

	Initial Assessment	6-Month Assessment	Annual Assessment	*p*
Age (years)	11.48 ± 0.25	11.97 ± 0.26 *	12.50 ± 0.25 *^,^**	*p* < 0.01
Body weight (kg)	65.27 ± 2.13	63.86 ± 2.20 *	64.16 ± 1.87 *	*p* < 0.01
Height (cm)	151.68 ± 1.31	154.29 ± 1.35 *	157.39 ± 1.20 *^,^**	*p* < 0.01
BMI (kg/m^2^)	27.55 ± 0.56	25.85 ± 0.62 *	25.33 ± 0.48 *	*p* < 0.01
BMI *z*-score	2.44 ± 0.15	1.80 ± 0.16 *	1.56 ± 0.13 *^,^**	*p* < 0.01
SBP (mmHg)	106.84 ± 1.22	106.43 ± 1.16	105.24 ± 1.09	NS
DBP (mmHg)	69.58 ± 0.89	67.14 ± 0.79 *	64.17 ± 0.76 *^,^**	*p* < 0.01
Waist circumference (cm)	90.39 ± 1.47	88.44 ± 1.79 *	86.18 ± 1.31 *	*p* < 0.01
Hip circumference (cm)	98.83 ± 1.47	96.6 ± 1.70 *	96.04 ± 1.16 *	*p* < 0.01
WHR	0.91 ± 0.01	0.91 ± 0.01	0.90 ± 0.01 *	*p* < 0.05
WHtR	0.59 ± 0.01	0.58 ± 0.01 *	0.55 ± 0.01 *^,^**	*p* < 0.01

Abbreviations: BMI, body mass index; BP, diastolic blood pressure; SBP, systolic blood pressure; WHR, waist-to-hip ratio; WHtR, waist-to-height ratio; all data are presented as mean ± standard error of mean. One-way ANOVA was used to compare all measurable variables. LSD post-hoc test indicated significant main effects. Statistical significance *p* < 0.05 and strong significance (*p* < 0.01) are presented. NS: nonsignificant difference (*p* > 0.05). *: indicates significant difference from initial assessment. **: indicates significant difference from 6-month assessment.

**Table 3:** (**A**) Biochemical parameters in all participants at initial and annual assessment. (**B**) Endocrinologic parameters in all participants at initial and annual assessment. (**C**) Body-composition parameters in all participants at initial and annual assessment.

(A)			
	Initial Assessment	Annual Assessment	*p*
Glucose (mg/dL)	91.24 ± 0.65	89.13 ± 0.66 *	<0.01
Urea (mg/dL)	27.88 ± 0.62	28.15 ± 0.59	NS
Creatinine (mg/dL)	0.58 ± 0.01	0.62 ± 0.01 *	<0.01
eGFR (mL/min/1.73 m^2^)	109.06 ± 1.82	110.15 ± 1.61	NS
AST (U/L)	23.20 ± 0.80	21.09 ± 0.83 *	<0.05
ALT (U/L)	23.18 ± 1.81	17.78 ± 0.87 *	<0.01
γGT (U/L)	14.34 ± 0.95	12.34 ± 0.48 *	<0.01
ALP (U/L)	254.01 ± 9.14	229.37 ± 9.64 *	<0.01
Phosphorus (mg/dL)	4.86 ± 0.05	4.87 ± 0.07	NS
Albumin (g/dL)	4.80 ± 0.02	4.84 ± 0.03	NS
Cholesterol (mg/dL)	157.83 ± 2.77	156.43 ± 2.36	NS
Triglycerides (mg/dL)	87.89 ± 5.63	80.24 ± 4.24 *	<0.05
HDL (mg/dL)	50.74 ± 1.13	53.30 ± 1.01 *	<0.01
LDL (mg/dL)	89.73 ± 2.54	87.37 ± 2.16	NS
Uric acid (mg/dL)	5.03 ± 0.11	4.98 ± 0.11	NS
Magnesium (mg/dL)	2.13 ± 0.01	2.10 ± 0.01	NS
K (mmol/L)	4.53 ± 0.03	4.46 ± 0.02 *	<0.05
Na (mmol/L)	140.23 ± 0.13	140.03 ± 0.15	NS
Ca (mmol/L)	9.98 ± 0.03	9.93 ± 0.03	NS
ApoA1 (mg/dL)	135.52 ± 2.28	134.23 ± 1.75	NS
ApoB (mg/dL)	79.59 ± 2.03	73.90 ± 1.67 *	<0.01
Lp(a) (mg/dL)	19.09 ± 2.55	22.39 ± 3.06 *	<0.01
(**B**)			
	**Initial Assessment**	**Annual Assessment**	***p***
TSH (μUI/mL)	2.74 ± 0.13	2.69 ± 0.13	NS
FT4 (mg/dL)	1.08 ± 0.01	1.14 ± 0.02 *	<0.01
T3 (ng/dL)	136.99 ± 2.05	133.73 ± 2.60	NS
Anti-TG (IU/mL)	30.02 ± 7.07	31.44 ± 9.25	NS
Anti-TPO (IU/mL)	32.45 ± 9.67	20.66 ± 4.15 *	<0.05
IGF-I (ng/mL)	223.07 ± 11.11	269.12 ± 9.01 *	<0.01
Androstenedione (ng/mL)	1.08 ± 0.10	1.28 ± 0.10 *	<0.01
Testosterone (ng/mL)	83.43 ± 10.19	134.09 ± 14.31 *	<0.01
DHEA-s (μg/dL)	130.26 ± 8.47	160.37 ± 9.86 *	<0.01
Prolactin (ng/mL)	9.50 ± 0.49	9.47 ± 0.54	NS
LH (mUI/mL)	2.45 ± 0.36	3.22 ± 0.49	NS
FSH (mUI/mL)	2.97 ± 0.20	3.50 ± 0.21 *	<0.01
E2 (pg/mL)	25.58 ± 1.78	35.23 ± 3.19 *	<0.01
PTH (pg/mL)	36.31 ± 1.33	40.63 ± 1.45 *	<0.01
25-OH-Vitamin D (ng/mL)	22.55 ± 0.67	25.07 ± 0.64 *	<0.01
ACTH (pg/mL)	26.02 ± 2.33	24.18 ± 1.25	NS
Cortisol (μg/dL)	13.21 ± 0.67	13.19 ± 0.46	NS
Insulin (μUI/mL)	21.97 ± 1.26	17.19 ± 0.83 *	<0.01
HbA1C (%)	5.33 ± 0.02	5.27 ± 0.03 *	<0.01
HOMA-IR	5.03 ± 0.30	3.83 ± 0.19 *	<0.01
(**C**)			
	**Initial Assessment**	**Annual Assessment**	***p***
Fat Percentage (%)	37.07 ± 0.84	31.99 ± 0.80 *	<0.01
Fat Mass (kg)	25.01 ± 1.29	21.37 ± 1.05 *	<0.01
Muscle Mass Percentage (%)	37.16 ± 1.03	40.60 ± 1.04 *	<0.01
Bone Mass (kg)	2.02 ± 0.05	2.20 ± 0.05 *	<0.01
Fat Free Mass (kg)	39.18 ± 1.08	42.80 ± 1.10 *	<0.01
Total Body Water	28.68 ± 0.79	31.33 ± 0.80 *	<0.01
Basal Metabolic Rate (Kilojoule)	6521.24 ± 117.91	6741.98 ± 116.65 *	<0.01
Impedance	691.21 ± 7.95	676.41 ± 8.06 *	<0.01
Phase Angle	5.38 ± 0.05	5.36 ± 0.05	NS

Abbreviations: ACTH, adrenocorticotropic hormone; ALP, alkaline phosphatase; ALT, alanine transaminase; anti-TG, antibodies against thyroglobulin; anti-TPO, thyroid peroxidase antibodies; ApoA1, apolipoprotein A1; ApoB, apolipoprotein B; AST, aspartate aminotransferase; DHEA-s, dehydroepiandrosterone sulfate; E2, estradiol; eGFR, estimated glomerular filtration rate; FSH, follicle stimulating hormone; FT4, free thyroxine; γGT, gamma-glutamyl transferase; HbA1C, hemoglobin A1C; HDL, high-density lipoprotein; HOMA-IR, homeostatic model assessment for insulin resistance; IGF-1, insulin-like growth factor 1; LDL, low-density lipoprotein; LH, luteinizing hormone; Lp(a), lipoprotein a; PTH, parathormone; T3, triiodothyronine; TSH, thyroid stimulating hormone; vitamin D, total 25-OH-vitamin D. All variables are presented as mean ± SE of mean. One-way ANOVA was used to compare all measurable variables. LSD post-hoc test indicated significant main effects. Statistical significance *p* < 0.05 and strong significance (*p* < 0.01) are presented. NS: nonsignificant difference (*p* > 0.05) *: indicates significant difference from initial assessment.

**Table 4:** Adipose tissue inflammation-associated parameters in all participants at initial and annual assessment.

	Initial Assessment	Annual Assessment	*p*
hs-CRP (mg/L)	4.02 ± 0.46	3.31 ± 0.40 *	<0.05
TAS (μmol/L)	253.97 ± 5.14	257.68 ± 4.95	NS
TOS (μmol/L)	788.55 ± 40.52	659.62 ± 40.21 *	<0.01
TNF-α (pg/mL)	0.99 ± 0.03	1.36 ± 0.13 *	<0.01
SFRP5 (ng/mL)	20.97 ± 1.12	18.70 ± 1.06 *	<0.01
IL-1α (pg/mL)	1.17 ± 0.14	1.21 ± 0.12	NS
IL-1β (pg/mL)	0.10 ± 0.01	0.18 ± 0.02 *	<0.01
IL-2 (pg/mL)	0.08 ± 0.01	0.12 ± 0.03	NS
IL-6 (pg/mL)	1.99 ± 0.11	1.80 ± 0.12	NS
IL-8 (pg/mL)	11.52 ± 0.56	14.40 ± 0.96 *	<0.01
IL-12 (pg/mL)	0.56 ± 0.02	0.02 ± 0.55	NS

Abbreviations: hs-CRP, high sensitivity *C*-reactive protein; SFRP5, secreted frizzled-related protein 5; IL, interleukin; TAS, total antioxidant status; TNF-α, tumor-necrosis factor-α; TOS; total oxidant status. All variables are presented as mean ± SE of mean. Participants were categorized as obese and overweight based on IOTF criteria at initial assessment. One-way ANOVA was used to compare all measurable variables. LSD post-hoc test indicated significant main effects. Statistical significance *p* < 0.05 and strong significance (*p* < 0.01) are presented. NS: nonsignificant difference (*p* > 0.05). *: indicates significant difference from initial assessment.

**Table 5:** (**A**) Clinical parameters of all participants at initial and annual assessment. (**B**) Biochemical parameters of all participants at initial and annual assessment. (**C**) Endocrinologic parameters of all participants at initial and annual assessment. (**D**) Adipose tissue inflammation-associated variables of all subjects at initial and annual assessment. (**E**) Body composition of all participants at initial and annual assessment.

(A)											
	Initial Assessment					Annual Assessment					*p*_between timepoints_
	Morbidly Obese	Obese	Overweight	Normal BMI	*p*_within_	Morbidly Obese	Obese	Overweight	Normal BMI	*p*_within_	
Age (years)	11.56 ± 0.37	11.14 ± 0.53	12.02 ± 0.52	10.75 ± 0.70	NS	12.59 ± 0.37 *	12.18 ± 0.54 *	13.03 ± 0.52 *	11.74 ± 0.69 *	NS	<0.01
Height (cm)	153.25 ± 1.96	153.19 ± 2.75	149.49 ± 1.98	145.80 ± 4.45	NS	158.43 ± 1.79 *	159.22 ± 2.67 *	155.95 ± 1.77 *	152.22 ± 4.20 *	NS	<0.01
BW (kg)	75.55 ± 3.11	63.31 ± 3.09 ^†^	54.30 ± 2.16 †	39.24 ± 3.49 ^†,‡ #^	<0.05	72.62 ± 2.75 *	63.20 ± 2.93	54.60 ± 1.90 ^†^	42.61 ± 3.42 *^,†,‡^	<0.05	<0.05
BMI (kg/m^2^)	31.22 ± 0.68	26.57 ± 0.44 ^†^	24.07 ± 0.40 ^†,‡^	17.95 ± 0.72 ^†,‡,#^	<0.05	28.27 ± 0.64 *	24.60 ± 0.43 *^,†^	22.32 ± 0.45 *^,†^	17.94 ± 0.66 ^†,‡,#^	<0.05	<0.05
BMI *z*-score	3.56 ± 0.16	2.12 ± 0.07 ^†^	1.31 ± 0.11 ^†,‡^	−0.33 ± 0.17 ^†,‡,#^	<0.01	2.44 ± 0.15 *	1.35 ± 0.08 *^,†^	0.54 ± 0.11 *^,†,‡^	−0.49 ± 0.15 ^†,‡,#^	<0.01	<0.05
Waist circumference (cm)	97.98 ± 1.89	91.76 ± 1.84 ^†^	81.79 ± 1.40 ^†,‡^	66.38 ± 2.79 ^†,‡,#^	<0.01	92.79 ± 1.70 *	88.07 ± 1.66 *	78.52 ± 1.44 *^,†,‡^	65.58 ± 2.44 ^†,‡,#^	<0.01	<0.01
Hip circumference (cm)	107.16 ± 1.94	96.18 ± 1.59 ^†^	89.55 ± 1.76 ^†^	79.42 ± 3.44 ^†,‡,#^	<0.01	101.87 ± 1.51 *	94.90 ± 1.83 ^†^	90.57 ± 1.53 ^†^	80.27 ± 3.13^†,‡^	<0.01	<0.05
WHR	0.91 ± 0.01	0.95 ± 0.01 ^†^	0.92 ± 0.02 ^‡^	0.84 ± 0.02 ^†,‡,#^	<0.01	0.91 ± 0.01	0.93 ± 0.01	0.87 ± 0.01 *^,†,‡^	0.82 ± 0.01 ^†,‡,#^	<0.01	<0.05
WHtR	0.64 ± 0.01	0.60±0.0.1 ^†^	0.55±0.01 ^†,‡^	0.45± 0.01^†,‡,#^	<0.01	0.59 ± 0.01 *	0.55 ± 0.01 *^,†^	0.50 ± 0.01 *^,†,‡^	0.43 ± 0.01 ^†,‡,#^	<0.01	<0.01
SBP (mmHg)	110.46 ± 1.52	107.49 ± 3.22	103.34 ± 2.54 ^†^	94.54 ± 3.09 ^†,‡,#^	<0.05	107.19 ± 1.54 *	107.02 ± 2.17	101.89 ± 2.25	98.73 ± 3.60 ^†^	<0.05	<0.05
DBP (mmHg)	73.47 ± 0.97	68.88 ± 2.09 ^†^	64.91 ± 2.16 ^†^	59.92 ± 2.04 ^†,‡^	<0.05	66.30 ± 1.08 *	62.69 ± 1.43 *	62.98 ± 1.78	58.28 ± 1.51 ^†^	<0.05	<0.05
(**B**)											
	**Initial Assessment**					**Annual assessment**					***p*_between timepoints_**
	**Morbidly Obese**	**Obese**	**Overweight**	**Normal BMI**	***p*_within_**	**Morbidly Obese**	**Obese**	**Overweight**	**Normal BMI**	***p*_within_**	
Ferritin (μg/L)	53.85 ± 4.34	54.71 ± 6.61	45.90 ± 6.14	48.29 ± 8.14	NS	52.90 ± 3.89	47.05 ± 6.33	42.00 ± 6.38	58.00 ± 15.38	NS	NS
Iron (μg/L)	79.88 ± 5.23	78.87 ± 8.74	83.35 ± 7.08	79.00 ± 9.03	NS	77.68 ± 4.20	81.83 ± 7.38	92.23 ± 8.17	84.57 ± 9.41 *	NS	<0.05
Glucose (mg/dL)	91.71 ± 0.83	91.52 ± 1.77	92.59 ± 1.72	86.57 ± 1.17 ^†,‡,#^	<0.05	88.73 ± 0.93 *	88.95 ± 1.60	90.55 ± 1.45	89.00 ± 2.02	NS	<0.05
Urea (mg/dL)	26.71 ± 0.78	27.43 ± 1.32	28.95 ± 1.52	32.14 ± 2.3 ^†,‡^	<0.05	28.10 ± 0.87	27.71 ± 1.05	27.14 ± 1.34	30.64 ± 1.90	NS	NS
Creatinine (mg/dL)	0.60 ± 0.02	0.56 ± 0.03	0.57 ± 0.02	0.57 ± 0.02	NS	0.63 ± 0.02 *	0.58 ± 0.03	0.61 ± 0.02 *	0.61 ± 0.02 *	NS	<0.05
eGFR (mL/min/1.73 m^2^)	108.36 ± 2.11	117.51 ± 5.16	110.75 ± 3.22	106.19 ± 3.43	NS	107.61 ± 2.42	118.79 ± 6.25 ^†^	107.49 ± 2.91 ^‡^	103.50 ± 2.71 ^‡^	<0.05	NS
AST (U/L)	22.25 ± 1.09	26.57 ± 2.46	22.68 ± 1.59	23.21 ± 1.74	NS	21.27 ± 1.48	21.10 ± 1.34 *	20.45 ± 1.05	21.2 ± 9 0.99	NS	<0.05
ALT (U/L)	22.71 ± 2.12	33.29 ± 6.72 ^‡^	19.91 ± 3.67 ^‡^	15.29 ± 1.84	<0.05	17.92 ± 1.33 *	20.95 ± 2.53 *	16.86 ± 1.27	13.86 ± 0.68	NS	<0.05
gGT (U/L)	14.41 ± 1.01	19.10 ± 4.31 ^†^	11.95 ± 0.67 ^‡^	10.64 ± 0.55 ^‡^	<0.05	12.22 ± 0.60 *	15.14 ± 1.84 *	10.82 ± 0.51	11.07 ± 0.63	NS	<0.05
ALP (U/L)	231.00 ± 13.29	292.29 ± 17.26 ^†^	270.64 ± 21.87	274.00 ± 21.25	<0.05	201.19 ± 12.33 *	266.90 ± 21.90 *^,†^	245.86 ± 23.41 *	278.69 ± 30.00 ^†^	<0.05	<0.01
Phosphorus (mg/dL)	4.79 ± 0.07	4.97 ± 0.09	4.98 ± 0.13	4.81 ± 0.14	NS	4.73 ± 0.11	5.10 ± 0.12 †	5.03 ± 0.14	4.90 ± 0.16	<0.05	NS
Cholesterol (mg/dL)	156.13 ± 3.39	163.81 ± 8.08	160.00 ± 7.96	153.07 ± 5.97	NS	158.30 ± 2.79	162.38 ± 7.02	151.32 ± 6.39 *	147.14 ± 6.27	NS	<0.05
Triglycerides (mg/dL)	99.05 ± 9.26	99.00 ± 11.73	64.91 ± 5.58 ^†,‡^	57.14 ± 7.64 ^†,‡^	<0.05	87.81 ± 6.87 *	91.57 ± 7.62	63.82 ± 6.21	55.00 ± 7.00 ^†,‡^	<0.05	<0.05
HDL (mg/dL)	47.83 ± 1.37	47.19 ± 1.68	55.77 ± 2.26 ^†,‡^	61.29 ± 4.90 ^†,‡^	<0.05	52.17 ± 1.41 *	50.38 ± 1.63 *	56.05 ± 2.29	58.43 ± 3.77 ^‡^	<0.05	<0.05
LDL (mg/dL)	88.78 ± 2.87	97.14 ± 7.07	91.25 ± 8.31	80.50 ± 4.86	NS	88.78 ± 2.49	94.19 ± 6.48	82.72 ± 6.33 *	78.14 ± 4.41	NS	<0.05
Uric acid (mg/dL)	5.29 ± 0.15	5.06 ± 0.27	4.82 ± 0.18	4.07 ± 0.29 ^†,‡^	<0.05	5.14 ± 0.15	5.04 ± 0.29	4.91 ± 0.19	4.23 ± 0.25 ^†^	<0.05	NS
Magnesium (mg/dL)	2.13 ± 0.02	2.13 ± 0.02	2.15 ± 0.03	2.07 ± 0.04	NS	2.10 ± 0.02	2.13 ± 0.02	2.07 ± 0.03	2.09 ± 0.03	NS	NS
K (mmol/L)	4.51 ± 0.03	4.56 ± 0.06	4.59 ± 0.07	4.50 ± 0.08	NS	4.44 ± 0.03	4.54 ± 0.06	4.47 ± 0.06	4.40 ± 0.09	NS	NS
Na (mmol/L)	140.33 ± 0.16	140.10 ± 0.29	140.55 ± 0.39	139.50 ± 0.23	NS	139.98 ± 0.20	139.52 ± 0.34	139.91 ± 0.36	141.14 ± 0.35 *^,†,‡,#^	<0.05	<0.05
Ca (mmol/L)	9.96 ± 0.04	10.05 ± 0.08	10.02 ± 0.06	9.92 ± 0.06	NS	9.92 ± 0.04	9.99 ± 0.07	9.92 ± 0.06	9.90 ± 0.06	NS	NS
ApoA1 (mg/dL)	133.30 ± 3.62	132.95 ± 3.27	137.91 ± 3.74	145.14 ± 7.42	NS	133.67 ± 2.66	134.22 ± 3.44	133.95 ± 3.36	136.93 ± 5.76	NS	NS
ApoB (mg/dL)	80.10 ± 2.30	87.20 ± 6.38 ^†^	77.77 ± 5.76 ^‡^	69.36 ± 4.00 ^‡^	<0.05	74.88 ± 2.09 *	82.39 ± 3.50 *	70.10 ± 5.11 *^,‡^	64.71 ± 3.07 ^‡^	<0.05	<0.01
Lp(a) (mg/dL)	16.42 ± 3.00	19.55 ± 6.72	22.78 ± 6.88	24.57 ± 9.26	NS	19.57 ± 3.89 *	23.01 ± 7.56	26.69 ± 8.37 *	26.37 ± 9.13	NS	<0.05
(**C**)											
	**Initial Assessment**					**Annual Assessment**					***p*_between timepoints_**
	**Morbidly Obese**	**Obese**	**Overweight**	**Normal BMI**	***p*_within_**	**Morbidly Obese**	**Obese**	**Overweight**	**Normal BMI**	***p*_within_**	
TSH (μUI/mL)	2.70 ± 0.18	3.14 ± 0.32	2.80 ± 0.29	2.26 ± 0.28	NS	2.92 ± 0.21	2.76 ± 0.30	2.15 ± 0.20 *^,†^	2.34 ± 0.23	<0.05	<0.05
FT4 (mg/dL)	1.07 ± 0.02	1.08 ± 0.04	1.07 ± 0.02	1.15 ± 0.03	NS	1.15 ± 0.02 *	1.17 ± 0.04 *	1.10 ± 0.03	1.15 ± 0.04	NS	<0.05
T3 (ng/dL)	135.27 ± 2.95	151.85 ± 4.42 ^†^	133.46 ± 4.25 ^‡^	129.07 ± 4.57 ^‡^	<0.05	129.94 ± 3.84	153.19 ± 5.39 ^†^	129.74 ± 4.89 ^‡^	127.85 ± 5.73 ^‡^	<0.05	NS
Anti-TG (IU/mL)	36.05 ± 13.41	31.93 ± 11.93	20.00	20.00	NS	36.98 ± 17.16	35.10 ± 16.07	20.00	20.00	NS	NS
Anti-TPO (IU/mL)	41.61 ± 18.06	34.72 ± 20.50	18.66 ± 2.67	16.66 ± 3.12	NS	22.49 ± 6.47	26.99 ± 13.76	15.12 ± 2.21	12.04 ± 1.03	NS	NS
IGF-1 (ng/mL)	225.91 ± 17.99	194.79 ± 19.70	240.81 ± 21.94	222.14 ± 26.72	NS	268.27 ± 13.27 *	273.00 ± 23.44 *	291.91 ± 16.80 *	231.86 ± 22.87 ^#^	<0.05	<0.05
Androstenedione (ng/mL)	1.28 ± 0.16	0.64 ± 0.08 ^†^	1.07 ± 0.22	0.82 ± 0.20	<0.05	1.50 ± 0.17 *	0.99 ± 0.17 *	1.10 ± 0.19	1.01 ± 0.21	NS	<0.05
Testosterone (ng/mL)	70.42 ± 11.11	61.69 ± 16.21	99.71 ± 28.84	150.14 ± 48.50 ^†^	<0.05	109.89 ± 16.71 *	107.09 ± 26.48 *	191.73 ± 41.84 *^,†,‡^	192.94 ± 55.19 ^†^	<0.05	<0.01
DHEA-s (μg/dL)	146.47 ± 13.08	130.21 ± 18.53	107.33 ± 13.07	93.40 ± 21.73	NS	173.67 ± 13.85 *	172.36 ± 26.71 *	138.85 ± 18.98 *	117.24 ± 26.56	NS	<0.01
Prolactin (ng/mL)	10.94 ± 0.82	8.18 ± 0.73	8.00 ± 0.71 ^†^	7.38 ± 0.67	<0.05	10.39 ± 0.95	8.87 ± 0.70	8.63 ± 0.60	7.52 ± 0.83	NS	NS
LH (mUI/mL)	2.94 ± 0.55	1.25 ± 0.29	2.76 ± 1.05	1.56 ± 0.39	NS	3.85 ± 0.88	2.10 ± 0.38	3.18 ± 0.53	2.08 ± 0.52	NS	NS
FSH (mUI/mL)	3.10 ± 0.30	2.47 ± 0.45	3.04 ± 0.43	3.06 ± 0.52	NS	3.66 ± 0.31 *	3.01 ± 0.55	3.68 ± 0.38	3.23 ± 0.65	NS	<0.05
E2 (pg/mL)	28.11 ± 3.19	23.68 ± 2.72	23.41 ± 2.50	21.26 ± 2.22	NS	41.50 ± 5.67 *	24.58 ± 2.77	33.53 ± 4.75	26.11 ± 2.40	NS	<0.05
PTH (pg/mL)	34.83 ± 1.49	34.97 ± 2.79	37.95 ± 2.27	40.85 ± 6.46	NS	39.21 ± 1.94 *	42.20 ± 3.31 *	40.99 ± 3.14 *	43.84 ± 5.38	NS	<0.05
Vitamin D (ng/mL)	22.18 ± 1.01	19.96 ± 0.90	22.20 ± 1.33	26.74 ± 1.79 ^†,‡,#^	<0.05	24.14 ± 0.90	22.98 ± 1.32 *	26.28 ± 1.27 *	29.66 ± 1.97 ^†,‡^	<0.05	<0.05
ACTH (pg/mL)	23.94 ± 2.56	30.64 8 ± 0.44	27.29 ± 7.27	26.05 ± 4.13	NS	23.45 ± 1.76	25.22 ± 3.30	26.02 ± 2.74	22.93 ± 3.48	NS	NS
Cortisol (μg/dL)	13.73 ± 1.12	11.23 ± 0.84	13.72 ± 1.28	13.10 ± 1.35	NS	13.45 ± 0.70	12.48 ± 1.00	13.14 ± 0.88	13.12 ± 1.34	NS	NS
Insulin (μUI/mL)	24.09 ± 1.71	29.08 ± 3.67	16.56 ± 1.79 ^†,‡^	10.25 ± 1.39 ^†,‡^	<0.05	18.10 ± 1.31 *	21.12 ± 1.37 *	15.38 ± 1.50	10.08 ± 1.04 ^†,‡^	<0.05	<0.05
HbA1c (%)	5.34 ± 0.03	5.39 ± 0.07	5.38 ± 0.05	5.17 ± 0.05 ^†,‡,#^	<0.05	5.26 ± 0.03 *	5.30 ± 0.07 *	5.35 ± 0.06	5.15 ± 0.06 ^#^	<0.05	<0.05
HOMA-IR	5.52 ± 0.42	6.63 ± 0.87	3.90 ± 0.47 ^†,‡^	2.21 ± 0.31 ^†,‡^	<0.05	4.00 ± 0.30 *	4.71 ± 0.36 *	3.51 ± 0.38	2.24 ± 0.25 ^†,‡^	<0.05	<0.05
(**D**)											
	**Initial Assessment**					**Annual Assessment**					***p*_between timepoints_**
	**Morbidly Obese**	**Obese**	**Overweight**	**Normal BMI**	***p*_within_**	**Morbidly Obese**	**Obese**	**Overweight**	**Normal BMI**	***p*_within_**	
Hs-CRP (mg/L)	4.56 ± 0.61	3.89 ± 0.76	3.69 ± 1.43	0.60 ± 0.23	NS	3.67 ± 0.56	2.60 ± 0.72	2.43 ± 0.94	4.17 ± 1.55	NS	NS
TAS (μmol/l)	256.06 ± 6.93	252.86 ± 16.67	244.69 ± 9.31	260.50 ± 12.10	NS	263.41 ± 6.59	251.96 ± 16.44	248.24 ± 8.85	255.00 ± 12.29	NS	NS
TOS (μmol/l)	874.76 ± 59.78	718.91 ± 77.01	823.47 ± 91.82	450.21 ± 70.33 ^†,#^	<0.05	695.21 ± 51.68 *	730.94 ± 128.35	635.83 ± 90.89 *	426.49 ± 69.27 ^†,‡^	<0.05	<0.05
TNF-α (pg/mL)	1.06 ± 0.05	1.02 ± 0.08	0.89 ± 0.07	0.81 ± 0.08	NS	1.23 ± 0.11	1.53 ± 0.43	1.60 ± 0.44 *	1.32 ± 0.28	NS	<0.05
SFRP5 (ng/mL)	21.92 ± 1.86	21.57 ± 2.98	20.03 ± 3.28	23.29 ± 3.40	NS	23.25 ± 4.75 *	18.61 ± 2.65	18.22 ± 3.30	19.64 ± 2.20	NS	<0.05
IL-1α (pg/mL)	1.02 ± 0.03	1.01 ± 0.01	1.88 ± 0.73 ^†,‡^	1.00 ± N/A	<0.05	1.09 ± 0.09	1.27 ± 0.27	1.55 ± 0.55	1.10 ± 0.08	NS	NS
IL-1β (pg/mL)	0.11 ± 0.01	0.10 ± 0.03	0.08 ± 0.01	0.08 ± 0.02	NS	0.17 ± 0.03	0.25 ± 0.04 *^,†^	0.20 ± 0.05 *	0.15 ± 0.03	<0.05	<0.05
IL-2 (pg/mL)	0.07 ± 0.01	0.13 ± 0.06	0.07 ± N/A	0.07 ± N/A	NS	0.08 ± 0.01	0.29 ± 0.18 *^,†^	0.07 ± N/A^*,‡^	0.16 ± 0.06	<0.05	<0.05
IL-6 (pg/mL)	2.21 ± 0.17	2.17 ± 0.22	1.55 ± 0.12 ^†^	1.40 ± 0.31 ^†^	<0.05	1.90 ± 0.15	1.92 ± 0.31	1.34 ± 0.20	1.90 ± 0.47	NS	NS
IL-8 (pg/mL)	11.26 ± 0.67	10.54 ± 0.72	12.87 ± 2.12	11.97 ± 1.32	NS	13.45 ± 1.02	13.88 ± 2.45	16.11 ± 3.29	16.95 ± 3.30 *	NS	<0.05
IL-12 (pg/mL)	0.56 ± 0.02	0.61 ± 0.05	0.52 ± 0.02	0.52 ± 0.02	NS	0.54 ± 0.02	0.57 ± 0.05	0.57 ± 0.04	0.50 ± N/A	NS	NS
(**E**)											
	**Initial Assessment**					**Annual Assessment**					***p*_between timepoints_**
	**Morbidly Obese**	**Obese**	**Overweight**	**Normal BMI**	***p*_within_**	**Morbidly Obese**	**Obese**	**Overweight**	**Normal BMI**	***p*_within_**	
Fat Percentage (%)	42.34 ± 0.85	37.09 ± 0.94 ^†^	32.05 ± 1.23 ^†,‡^	21.23 ± 1.36 ^†,‡,#^	<0.01	36.65 ± 0.92 *	32.10 ± 1.21 *^,†^	26.29 ± 1.39 *^,†,‡^	19.79 ± 1.12 ^†,‡,#^	<0.01	<0.01
Fat Mass (kg)	32.14 ± 1.87	22.81 ± 1.06 ^†^	17.09 ± 1.11 ^†^	8.49 ± 1.20 ^†,‡,#^	<0.01	27.12 ± 1.53 *	19.97 ± 0.84 *^,†^	14.38 ± 0.98 *^,†^	8.58 ± 1.03 ^†,‡^	<0.01	<0.01
Muscle Mass Percentage (%)	39.98 ± 1.55	37.24 ± 2.16	34.28 ± 1.47 ^‡^	28.85 ± 2.52 ^†,‡^	<0.05	43.13 ± 1.57 *	41.09 ± 2.33 *	38.20 ± 1.47 *	32.24 ± 2.52 *^,†,‡^	<0.05	<0.01
Bone Mass (kg)	2.16 ± 0.08	2.04 ± 0.11	1.87 ± 0.07 ^†^	1.62 ± 0.13 ^†,‡^	<0.05	2.32 ± 0.08 *	2.22 ± 0.12 *	2.07 ± 0.07 *	1.79 ± 0.13 *^,†,‡^	<0.05	<0.01
Fat Free Mass (kg)	42.14 ± 1.63	39.27 ± 2.27	36.15 1 ± 0.55 ^†^	30.47 ± 2.65 ^†,‡^	<0.05	45.45 ± 1.65 *	43.31 ± 2.44 *	40.27 ± 1.54 *	34.03 ± 2.65 *^,†,‡^	<0.05	<0.01
Total Boby Water	30.85 ± 1.19	28.75 ± 1.66	26.46 ± 1.13 ^†^	22.30 ± 1.93 ^†,‡^	<0.05	33.28 ± 1.21 *	31.70 ± 1.79 *	29.48 ± 1.13 *	24.91 ± 1.94 *^,†,‡^	<0.05	<0.01
Basal Metabolic Rate (Kilojoule)	6910.33 ± 176.44	6599.70 ± 238.03	6013.27 ± 140.20 ^†^	5456.50 ± 254.59 ^†,‡^	<0.05	7089.25 ± 176.94 *	6872.52 ± 244.57 *	6268.45 ± 133.01 *^,†^	5727.57 ± 250.69 *^,†,‡^	<0.05	<0.01
Impedance	662.26 ± 10.30	705.25 ± 20.09 ^†^	722.01 ± 16.12 ^†^	753.03 ± 19.34 ^†^	<0.05	655.02 ± 10.24	700.17 ± 24.06 ^†^	683.47 ± 17.91 *	727.61 ± 17.53 *^,†^	<0.05	<0.05
Phase Angle	5.44 ± 0.06	5.30 ± 0.14	5.30 ± 0.10	5.30 ± 0.10	NS	5.37 ± 0.07	5.38 ± 0.15	5.30 ± 0.12	5.35 ± 0.13	NS	NS

Abbreviations: ACTH, adrenocorticotropic hormone; ALP, alkaline phosphatase; ALT, alanine transaminase; anti-TG, antibodies against thyroglobulin; anti-TPO, thyroid peroxidase antibodies; apoA1, apolipoprotein A1; apoB, apolipoprotein B; AST, aspartate aminotransferase; BMI, body mass index; BW, body weight; DBP, diastolic blood pressure; DHEA-s, dehydroepiandrosterone sulfate; E2, estradiol; eGFR, estimated glomerular filtration rate; FSH, follicle stimulating hormone; FT4, free thyroxine; hs-CRP, high sensitivity *C*-reactive protein; γGT, gamma-glutamyl transferase; HbA1C, hemoglobin A1C; HDL, high-density lipoprotein; HOMA-IR, homeostatic model assessment for insulin resistance; IGF-1, insulin-like growth factor 1; IL, interleukin; LDL, low-density lipoprotein; LH, luteinizing hormone; Lp(a), lipoprotein a; PTH, parathormone; SBP, systolic blood pressure; SFRP5, secreted frizzled related protein 5; T3, triiodothyronine; TAS, total antioxidant status; TNF-α, tumor-necrosis factor-a; TOS; total oxidant status; TSH, thyroid stimulating hormone; vitamin D, total 25-OH-vitamin D; WHR, waist-to-hip ratio; WHtR, waist-to-height ratio. All variables are presented as mean ± SE of mean. One-way ANOVA was used to compare all measurable variables. LSD post-hoc test indicated significant main effects. Statistical significance *p* < 0.05 and strong significance (*p* < 0.01) are presented. NS: nonsignificant (*p* > 0.05) difference. *: indicates significant difference from initial assessment. ^†^: indicates significant difference from morbid obesity. ^‡^: indicates significant difference from obese. ^#^: Indicates significant difference from overweight. N/A: not applicable.

## Data Availability

The corresponding author can provide the data from this study upon request. Because of privacy restrictions, the data are not accessible to the public.

## Supplementary Materials

The following supporting information can be downloaded at https://www.mdpi.com/article/10.3390/nu16183133/s1, Supplemental Table S1: correlation coefficient analysis between assessed variables and Sfrp5 concentrations in the morbid obesity, obesity, overweight, and normal BMI groups at initial assessment. Supplemental Table S2: predictors of Sfrp5 and ΔSfrp5. Supplemental Figures S1–S23: normality plots of the most relevant variables.
