# Supplementary material for: The Role of Secreted Frizzled-Related Protein 5 (Sfrp5) in Overweight and Obesity in Childhood and Adolescence
Source: Nutrients. 2024 Sep 17;16(18):3133. doi: 10.3390/nu16183133 (PMC11434931; doi:10.3390/nu16183133)
Supplement: Supplementary file 1 [file nutrients-16-03133-s001.zip › nutrients-3131026_Supplemental figures_Normality plots.pdf]

SUPPLEMENTAL FIGURES

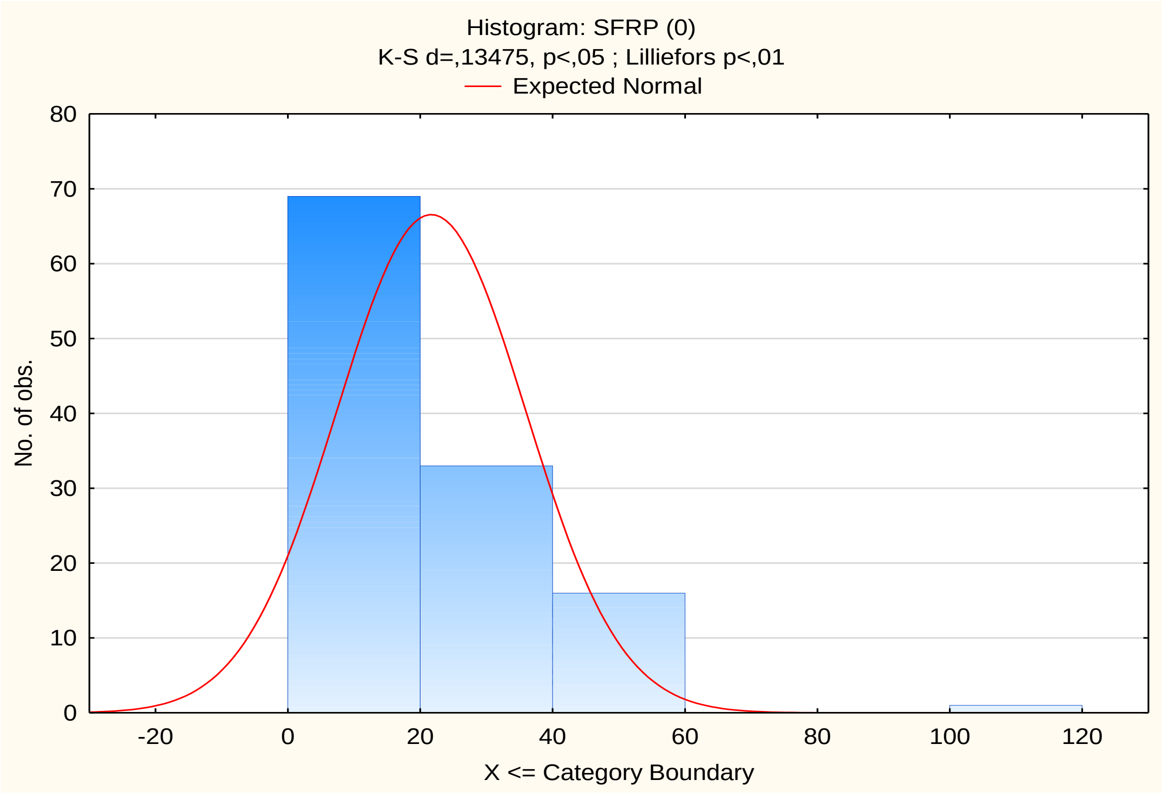

Figure S1. Normality plot of initial concentration of Sfrp5.

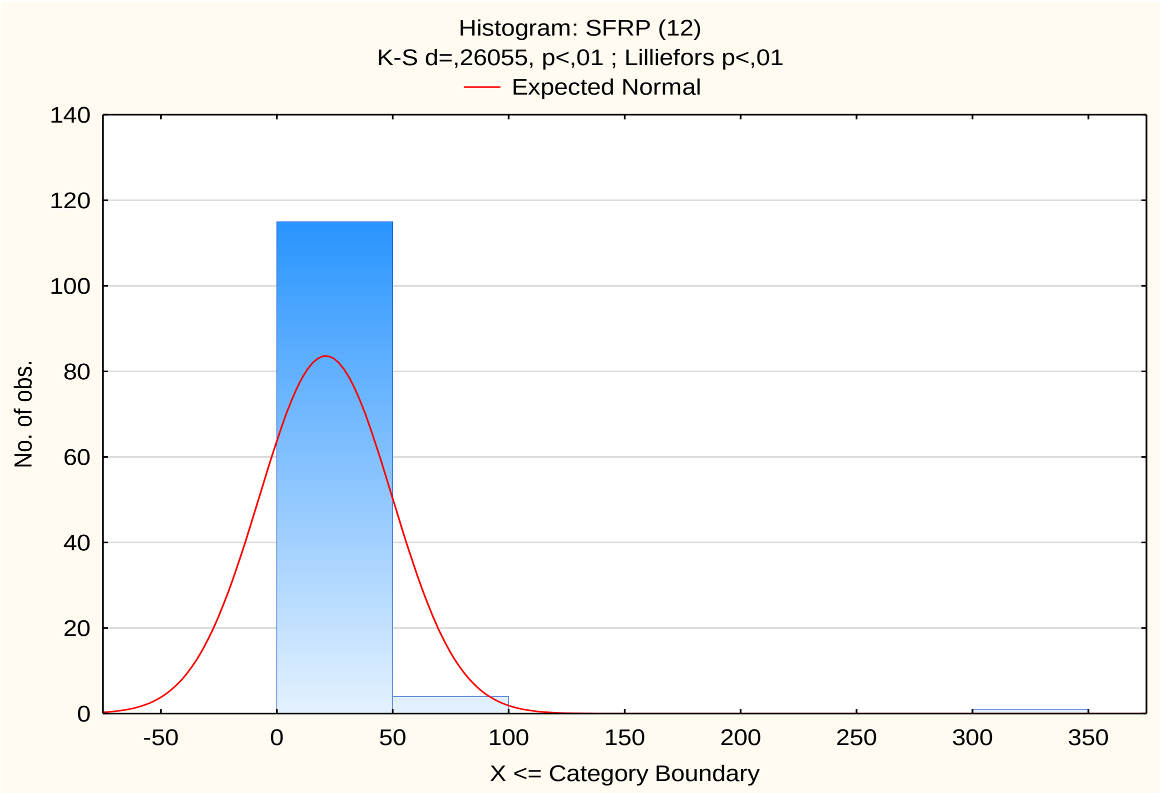

Figure S2. Normality plot of annual concentration of Sfrp5.

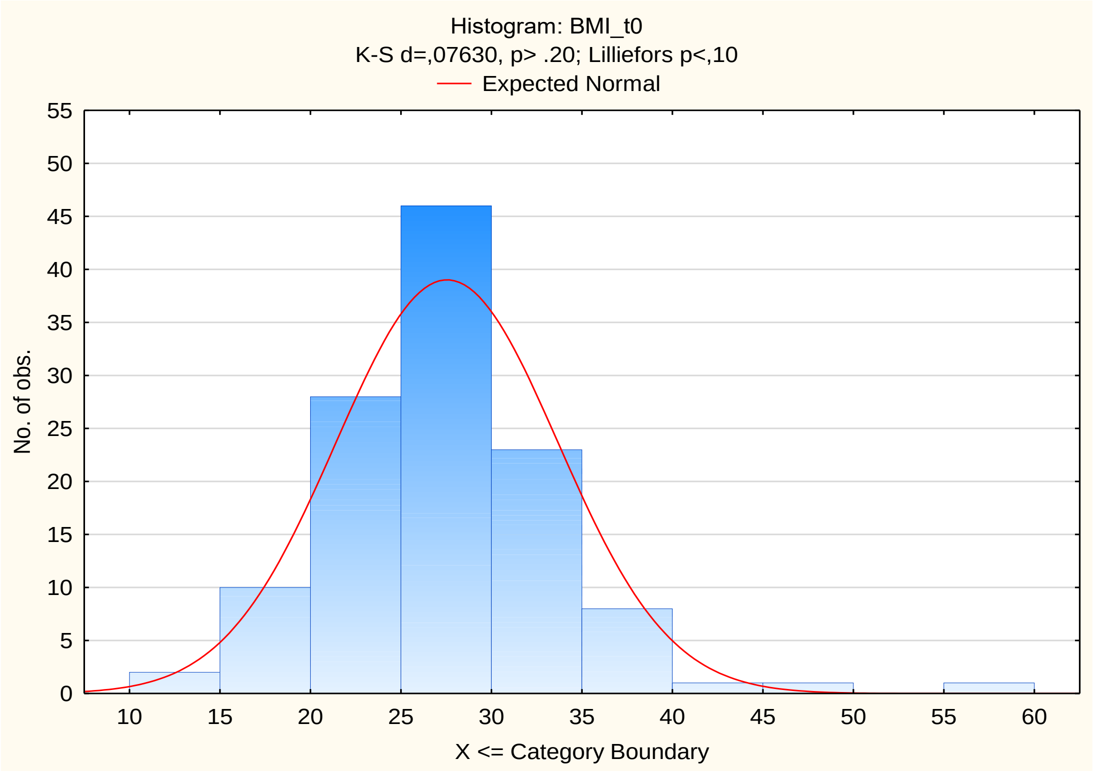

Figure S3. Normality plot of initial BMI.

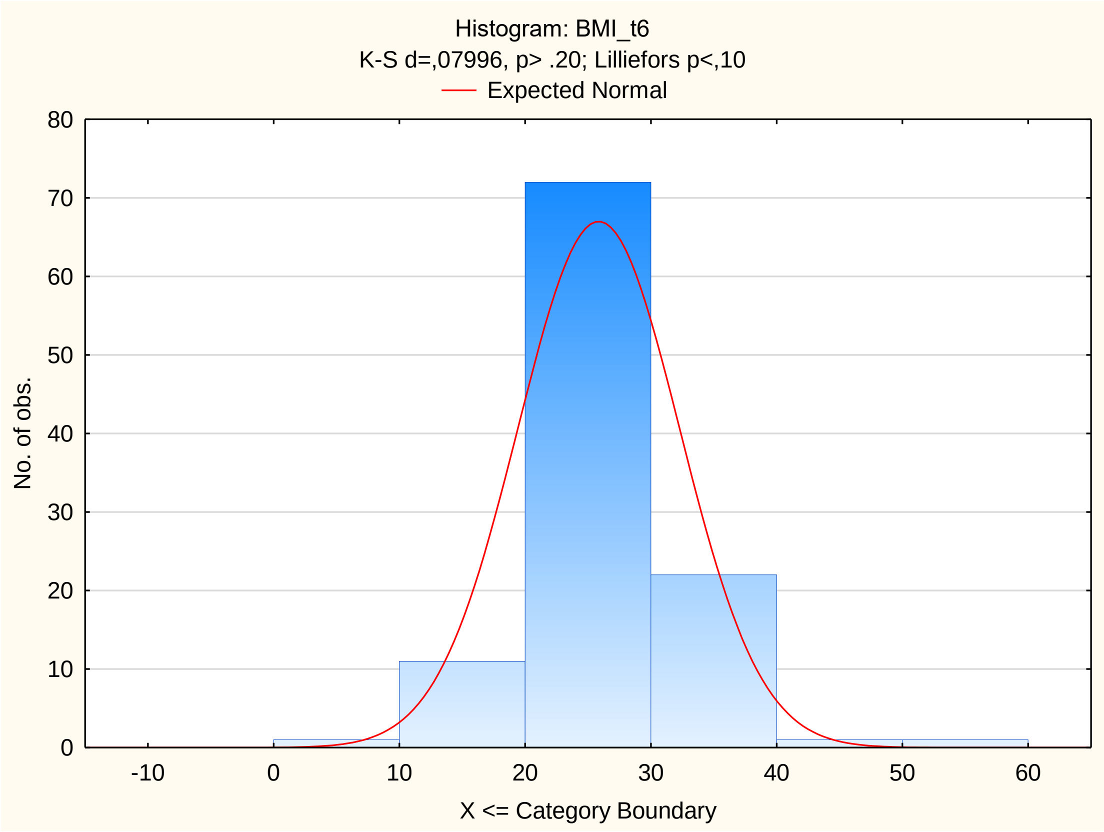

Figure S4. Normality plot of BMI at 6 months.

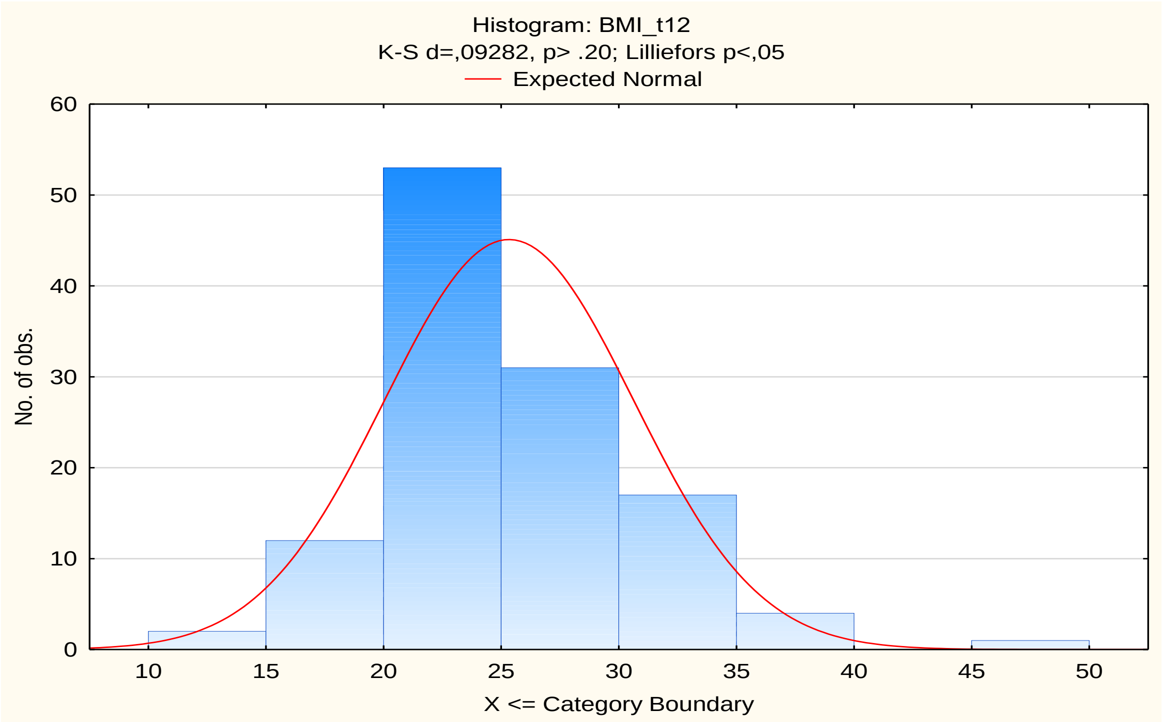

Figure S5. Normality plot of annual BMI.

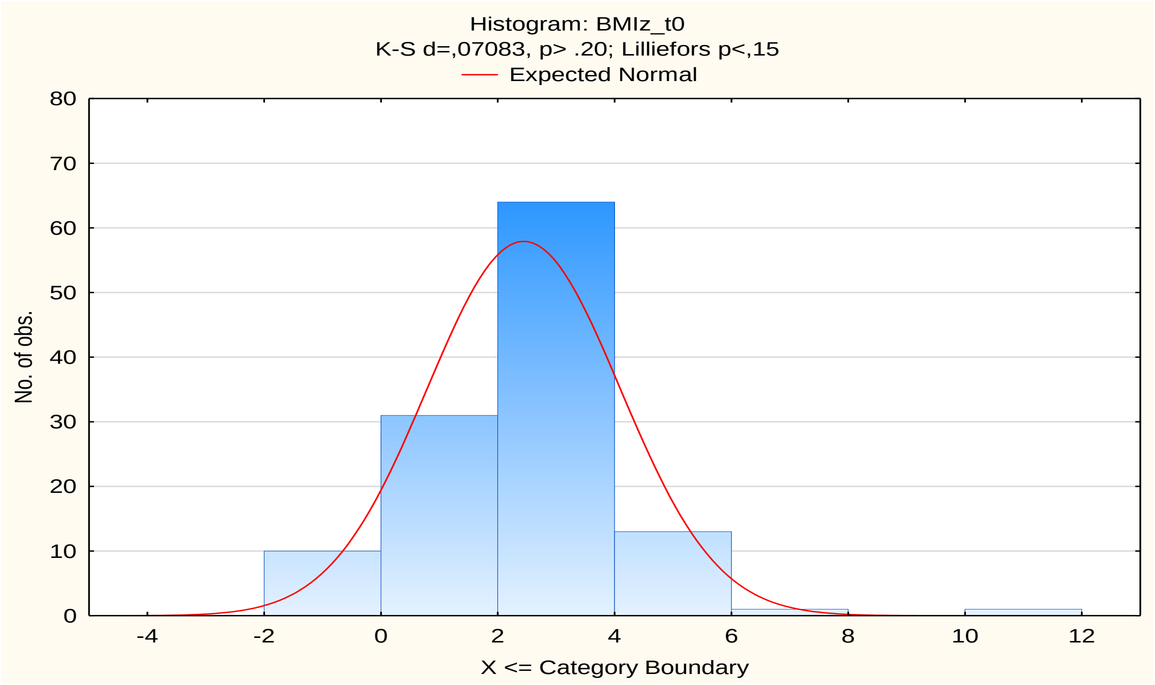

Figure S6. Normality plot of initial BMI z-score.

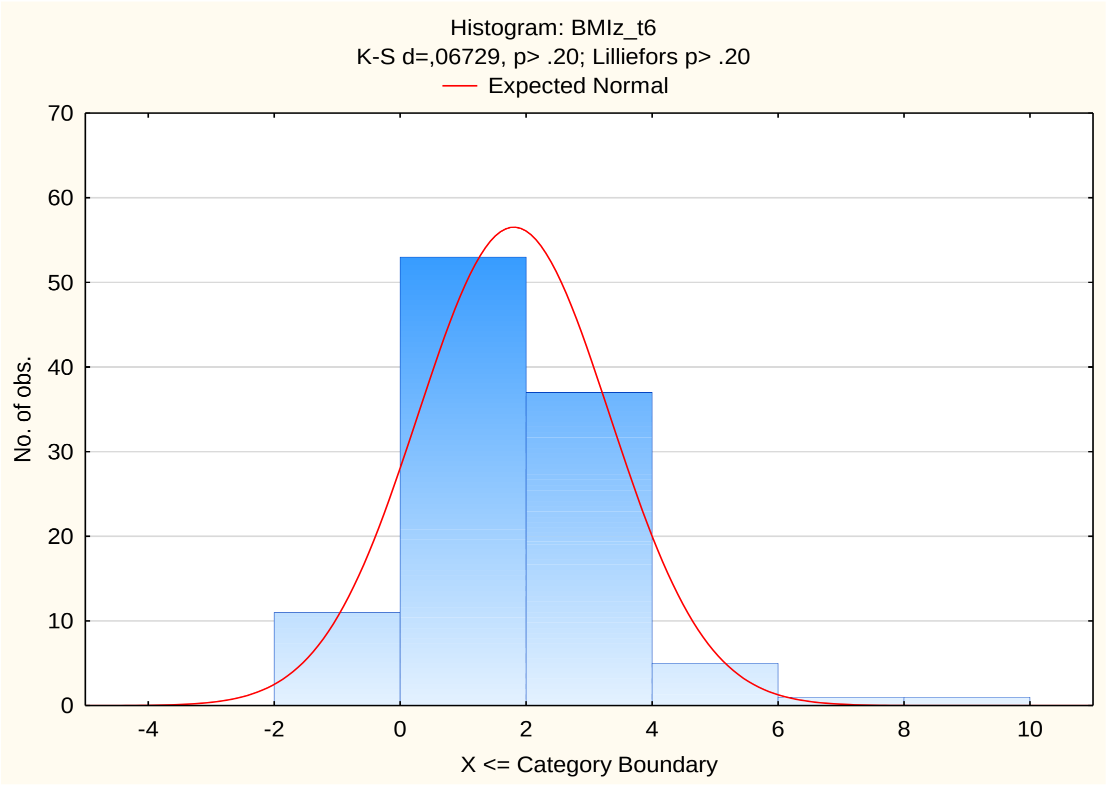

Figure S7. Normality plot of BMI z-score at 6 months.

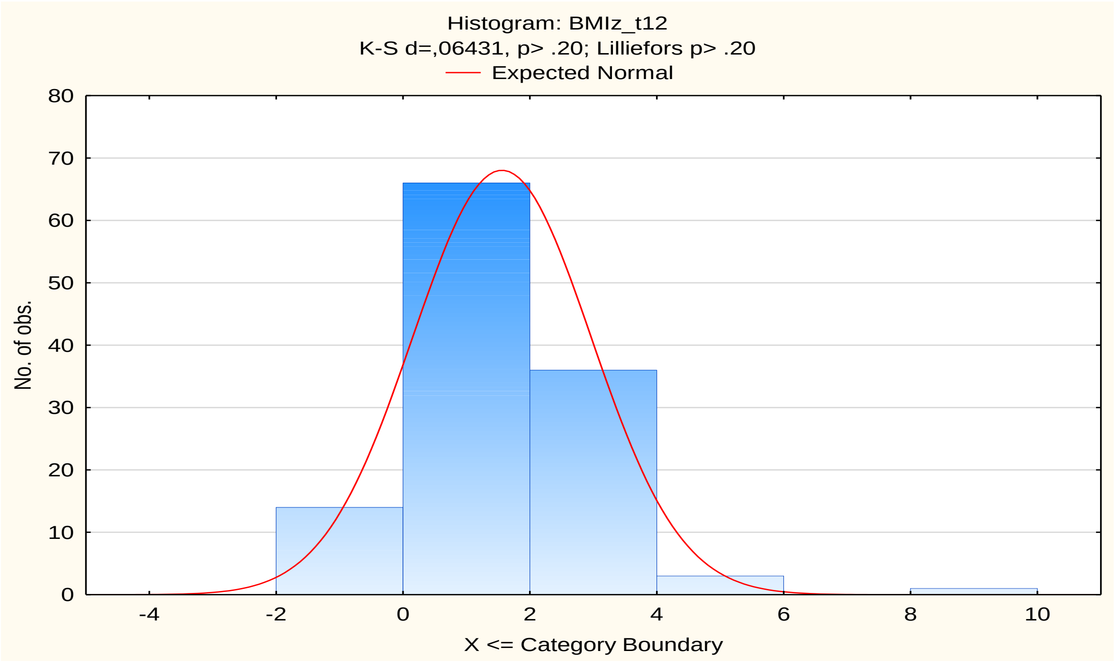

Figure S8. Normality plot of annual BMI z-score.

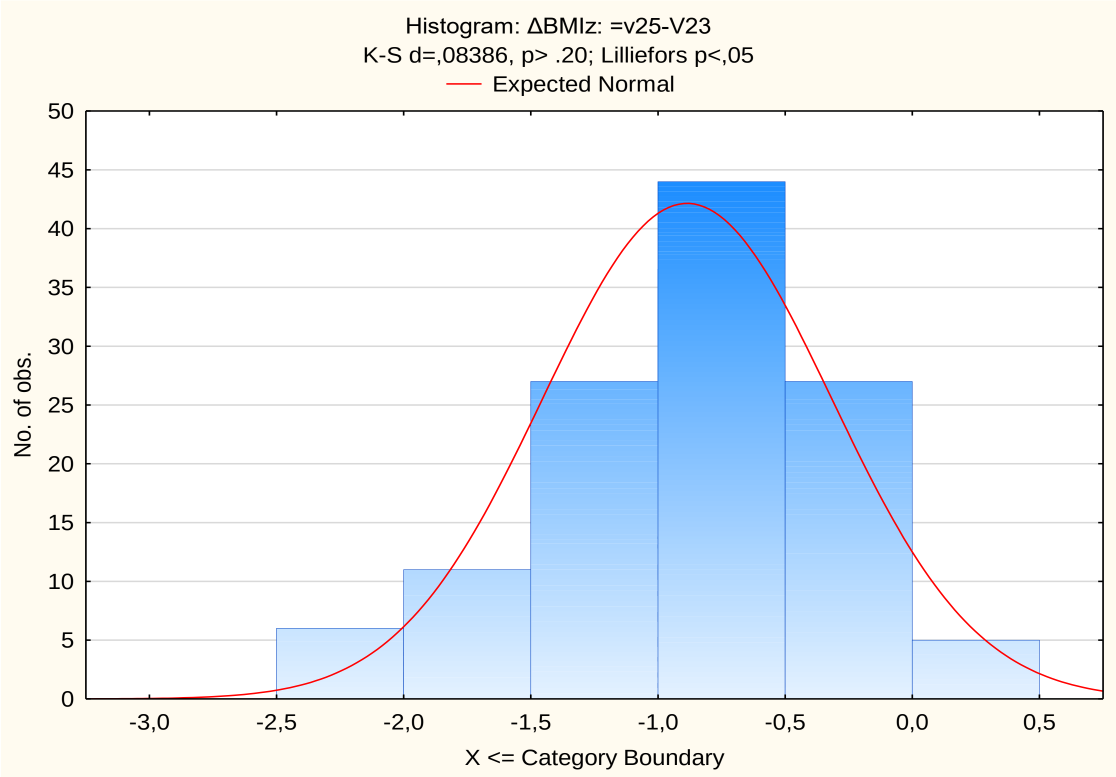

Figure S9. Normality plot of the change of BMI z-score (ΔBMIz-score).

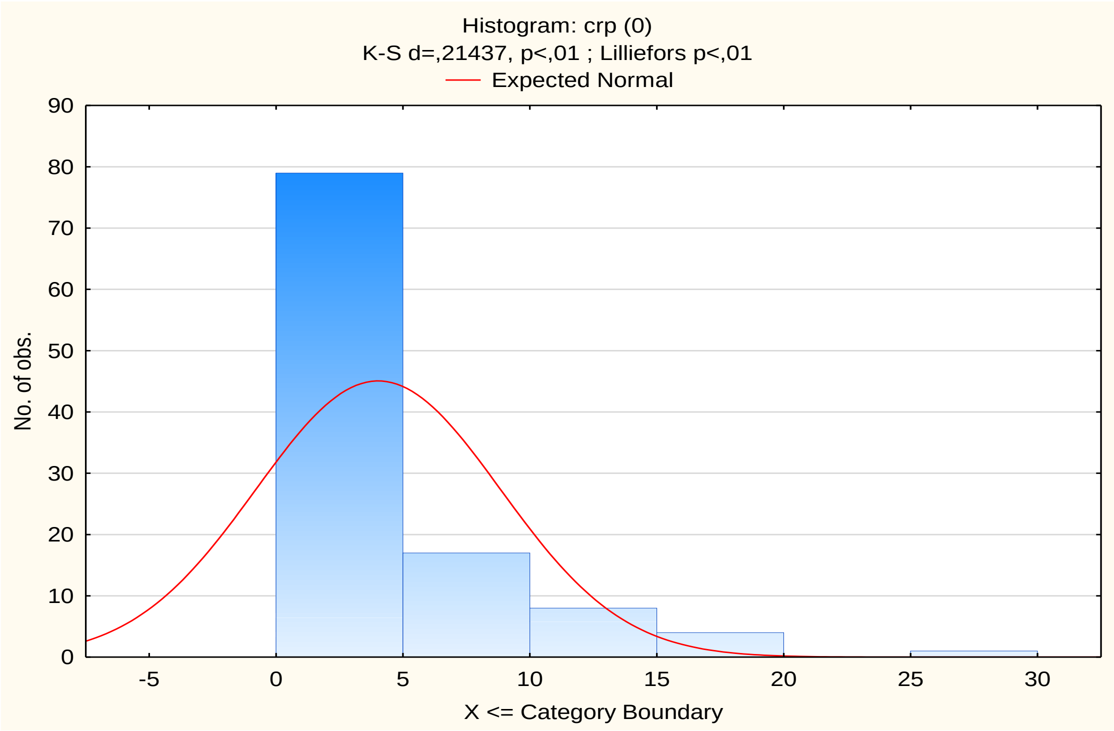

Figure S10. Normality plot of initial concentration of hs-CRP.

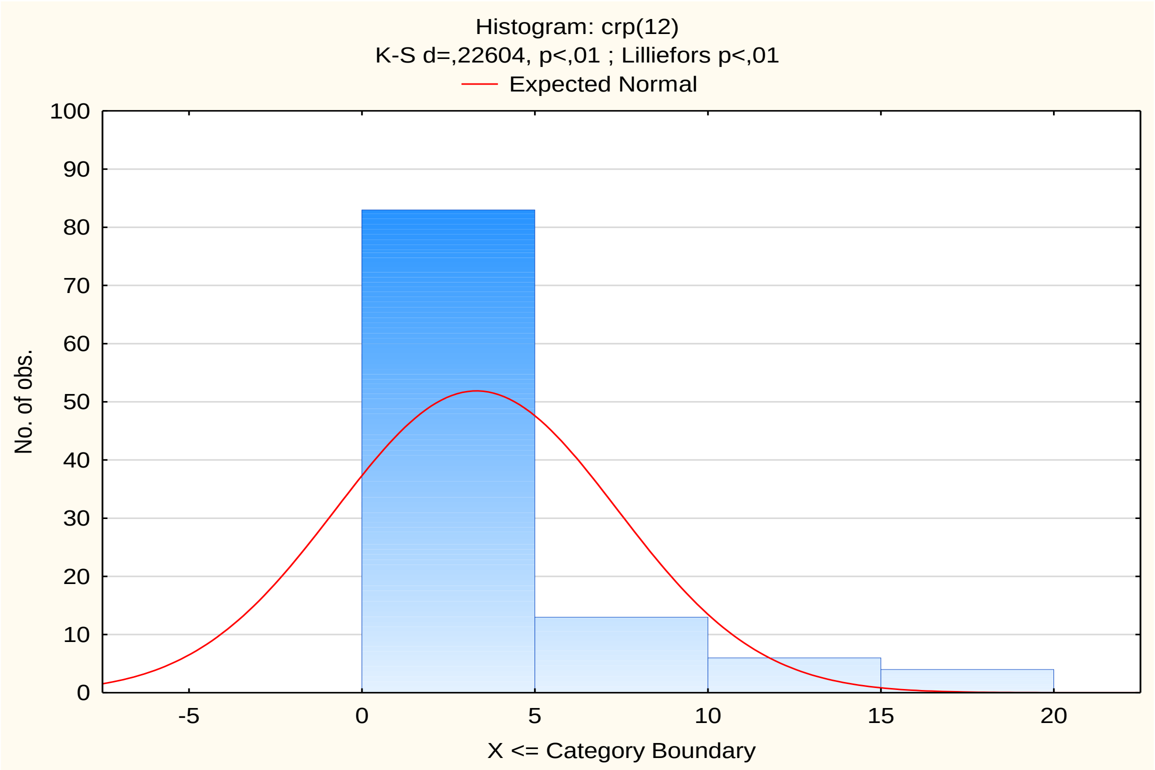

Figure S11. Normality plot of annual concentration of hs-CRP.

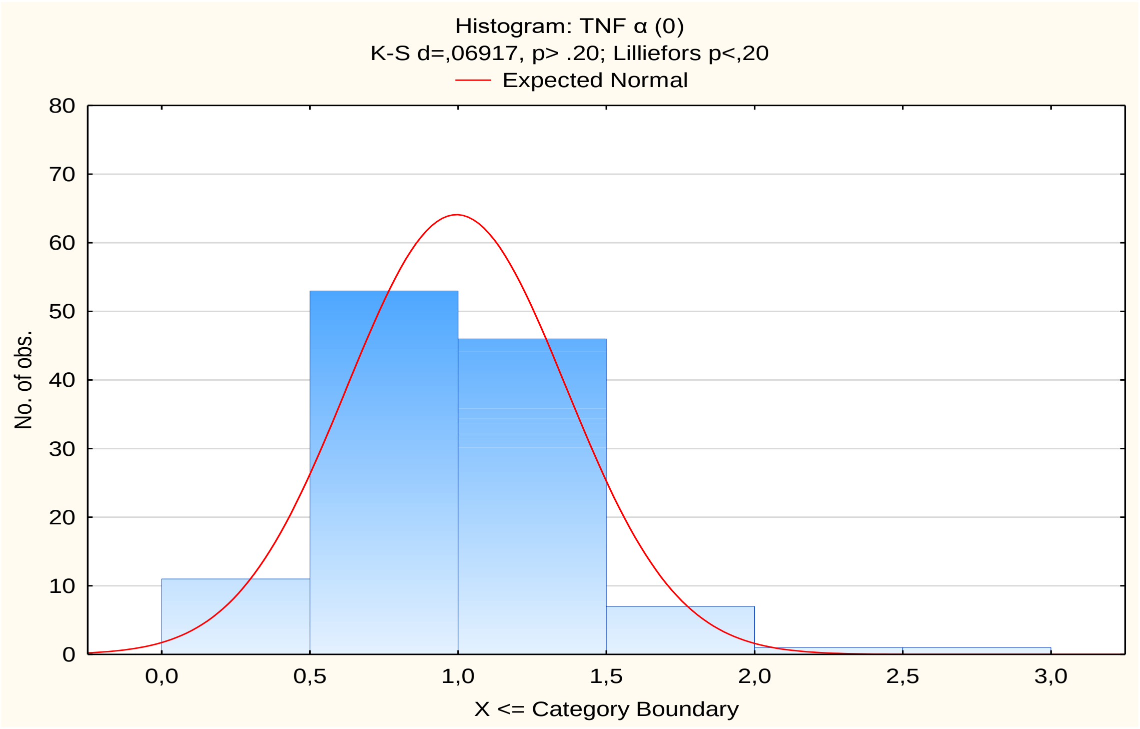

Figure S12. Normality plot of initial concentration of TNF-a.

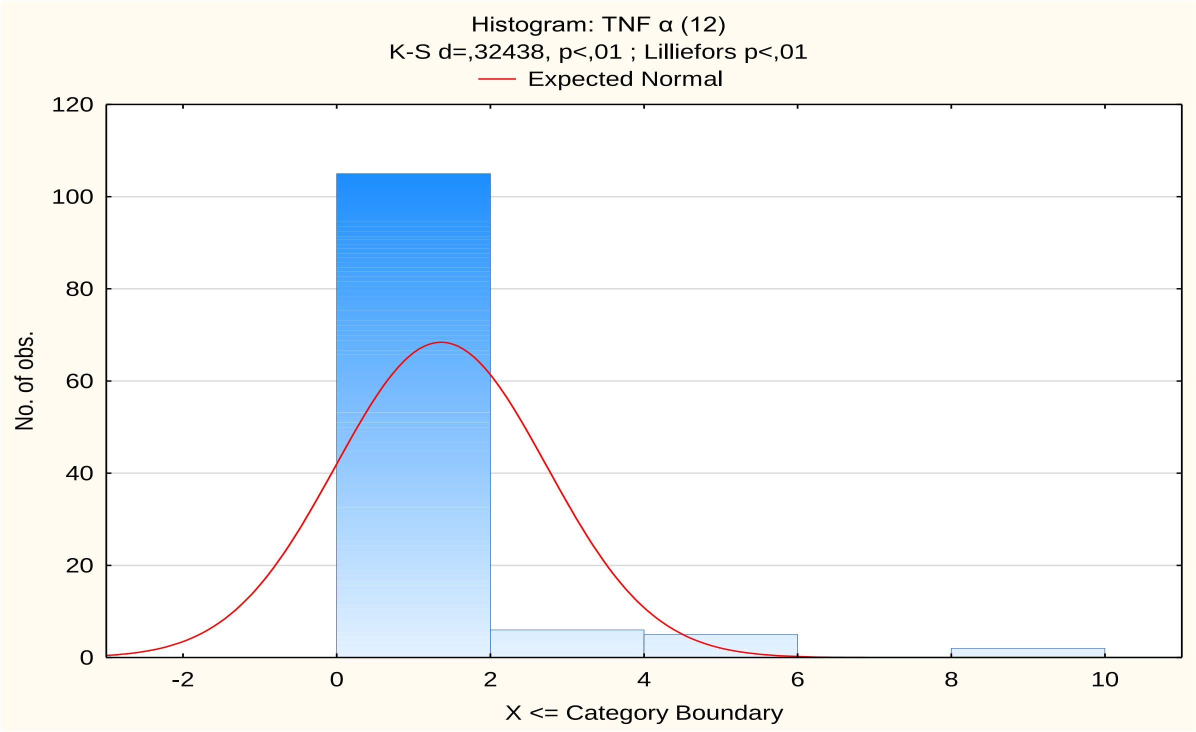

Figure S13. Normality plot of annual concentration of TNF-a.

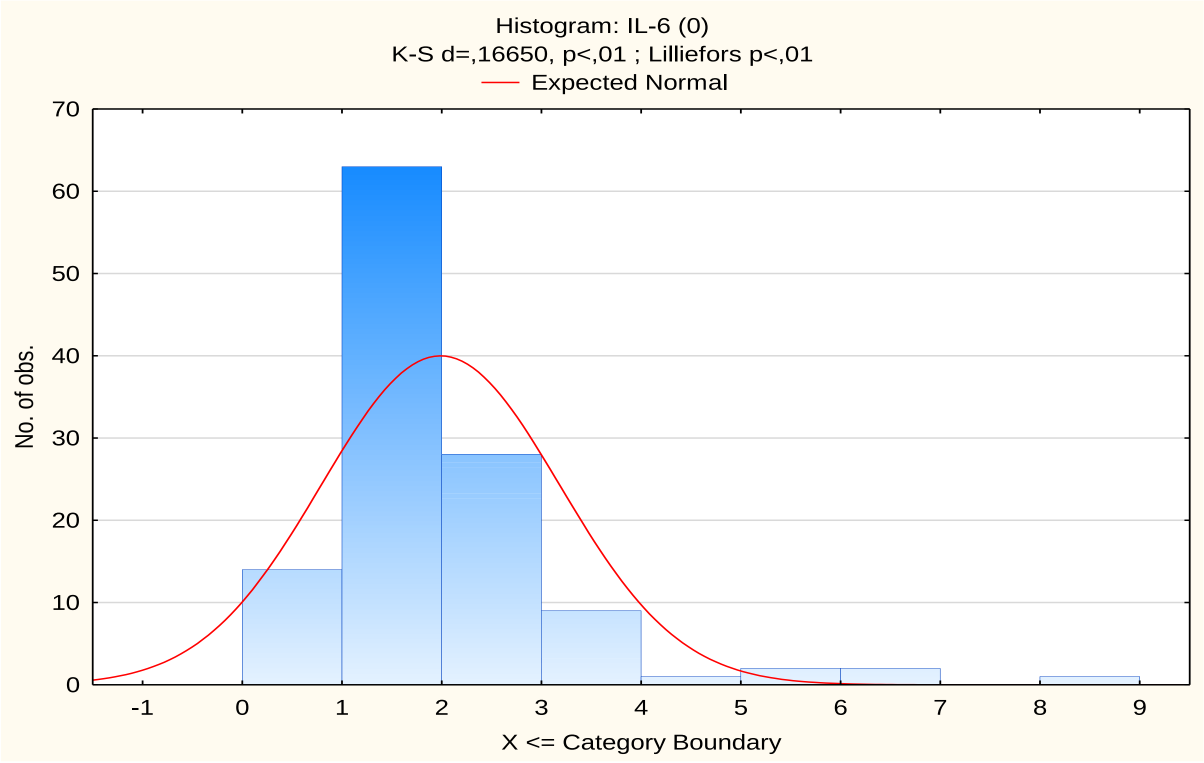

Figure S14. Normality plot of initial concentration of IL-6.

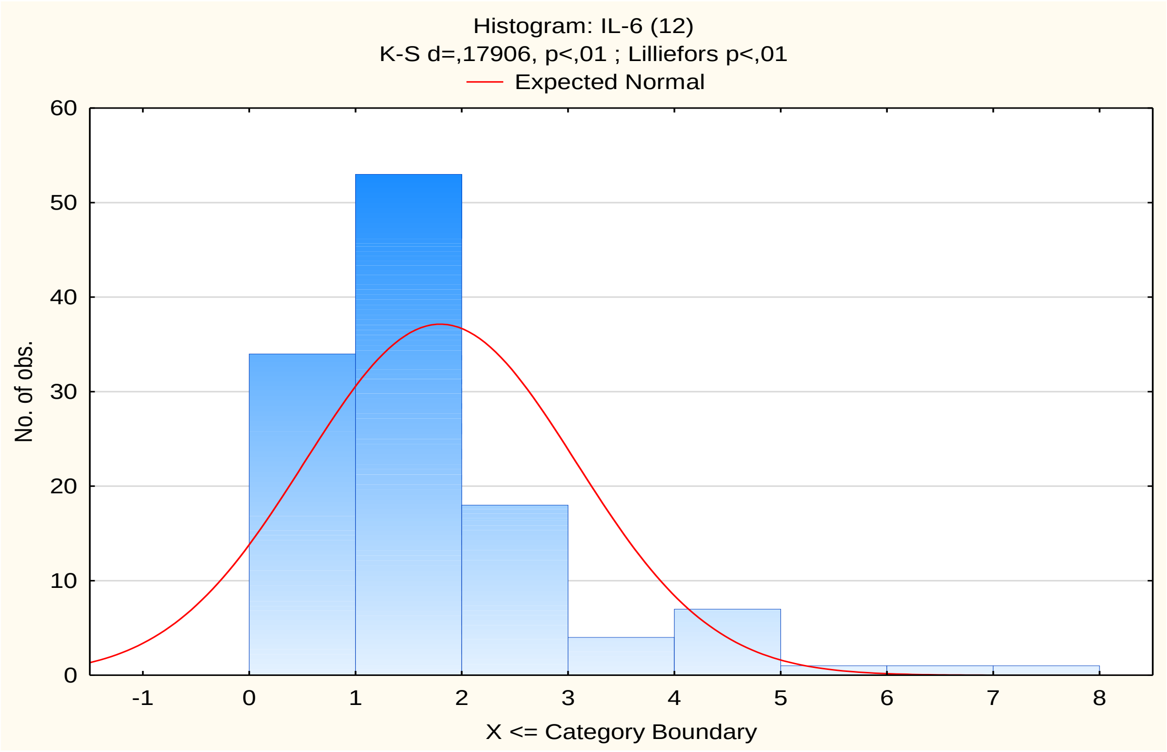

Figure S15. Normality plot of annual concentration of IL-6.

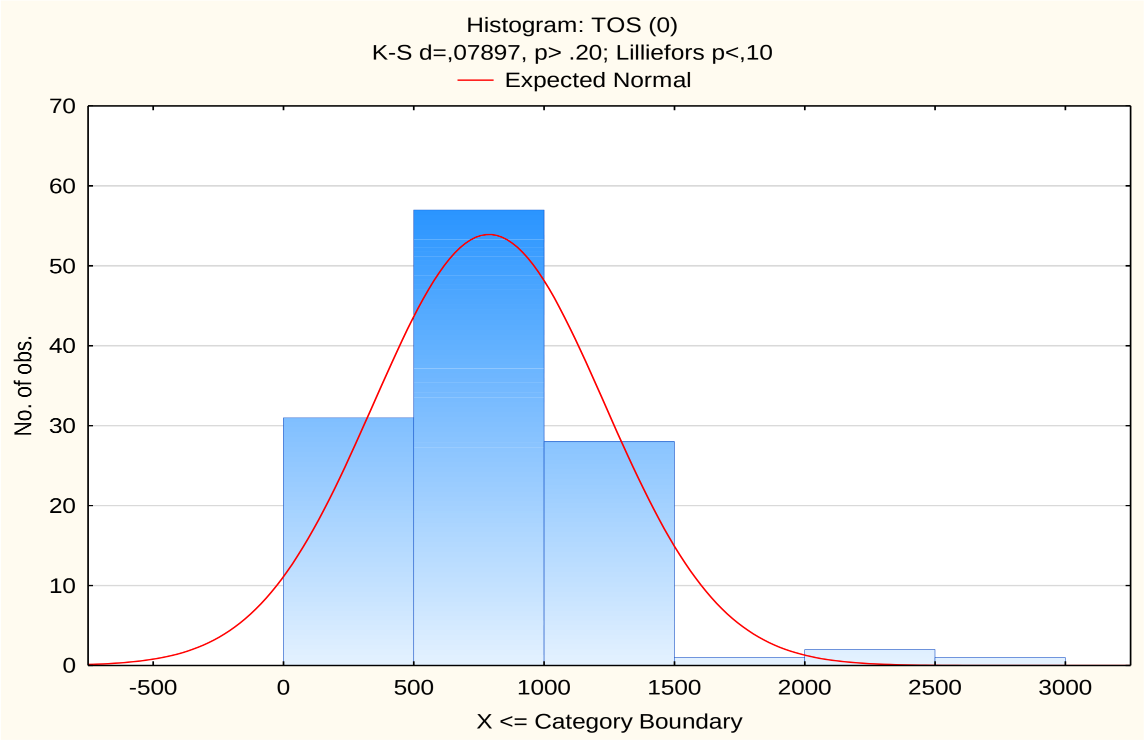

Figure S16. Normality plot of initial concentration of Total Oxidative Status.

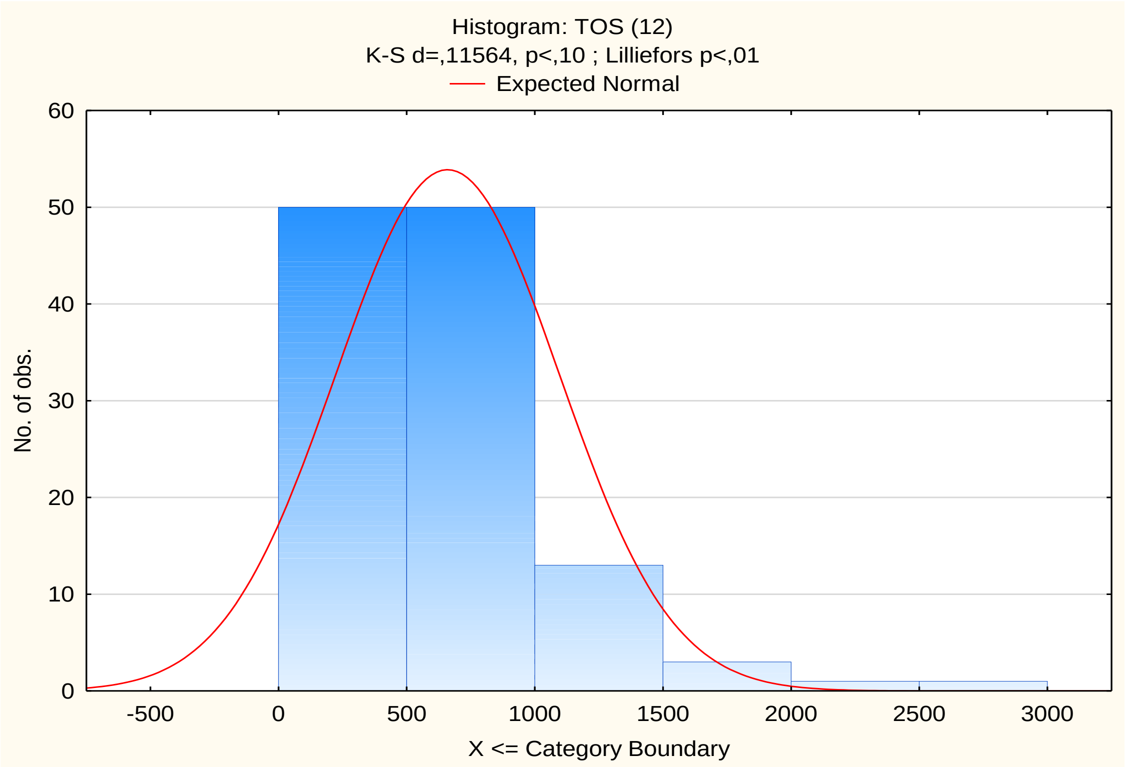

Figure S17. Normality plot of annual concentration of Total Oxidative Status.

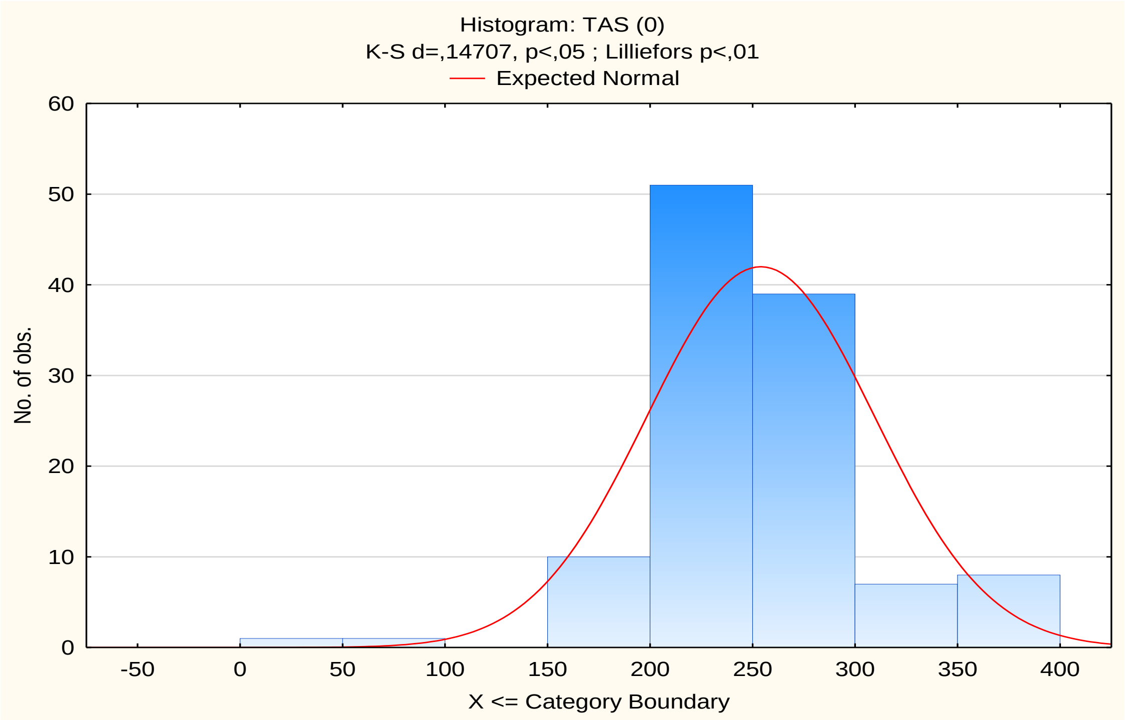

Figure S18. Normality plot of initial concentration of Total Antioxidative Status.

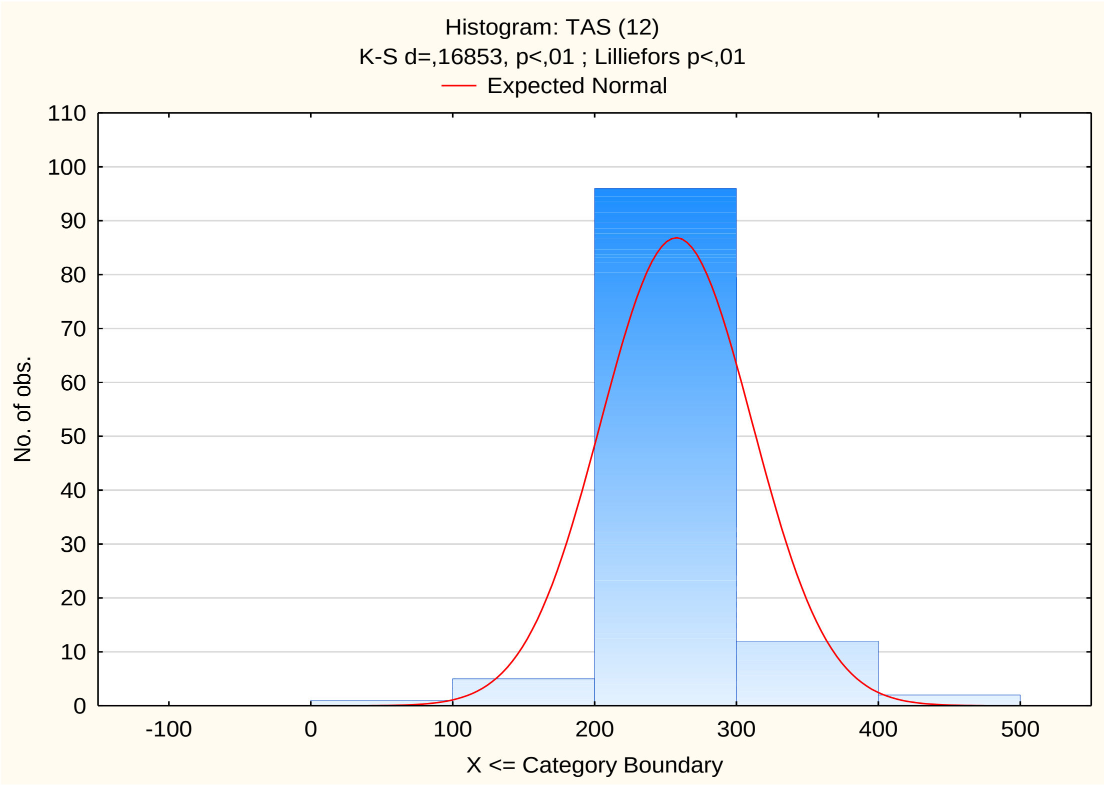

Figure S19. Normality plot of annual concentration of Total Antioxidative Status.

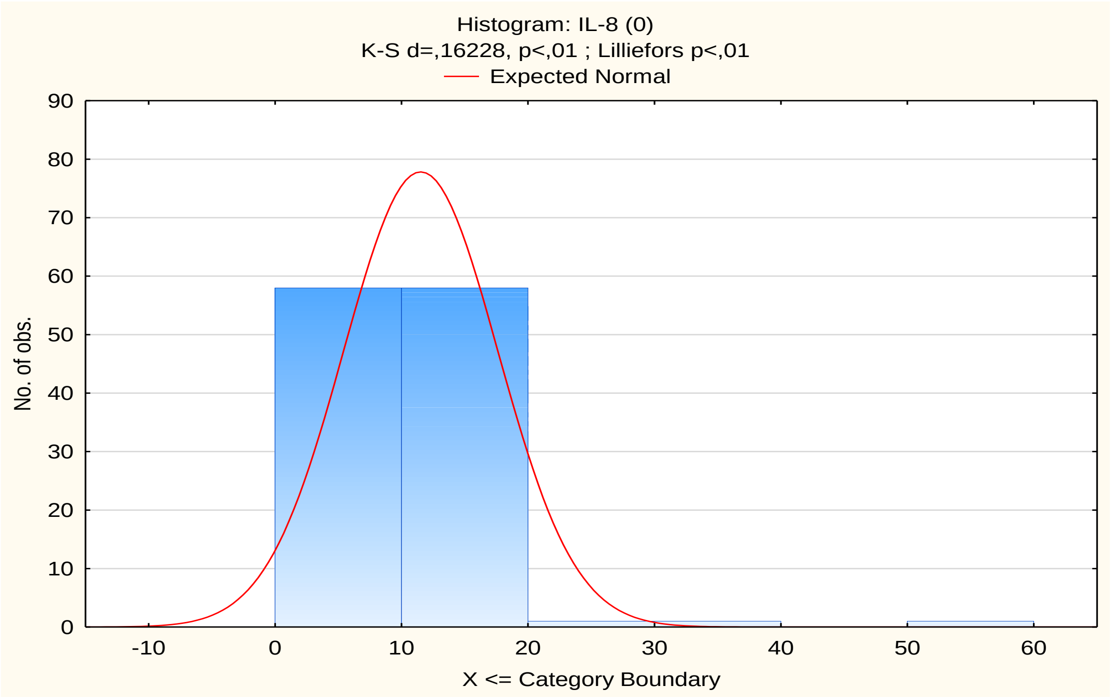

Figure S20. Normality plot of initial concentration of IL-8.

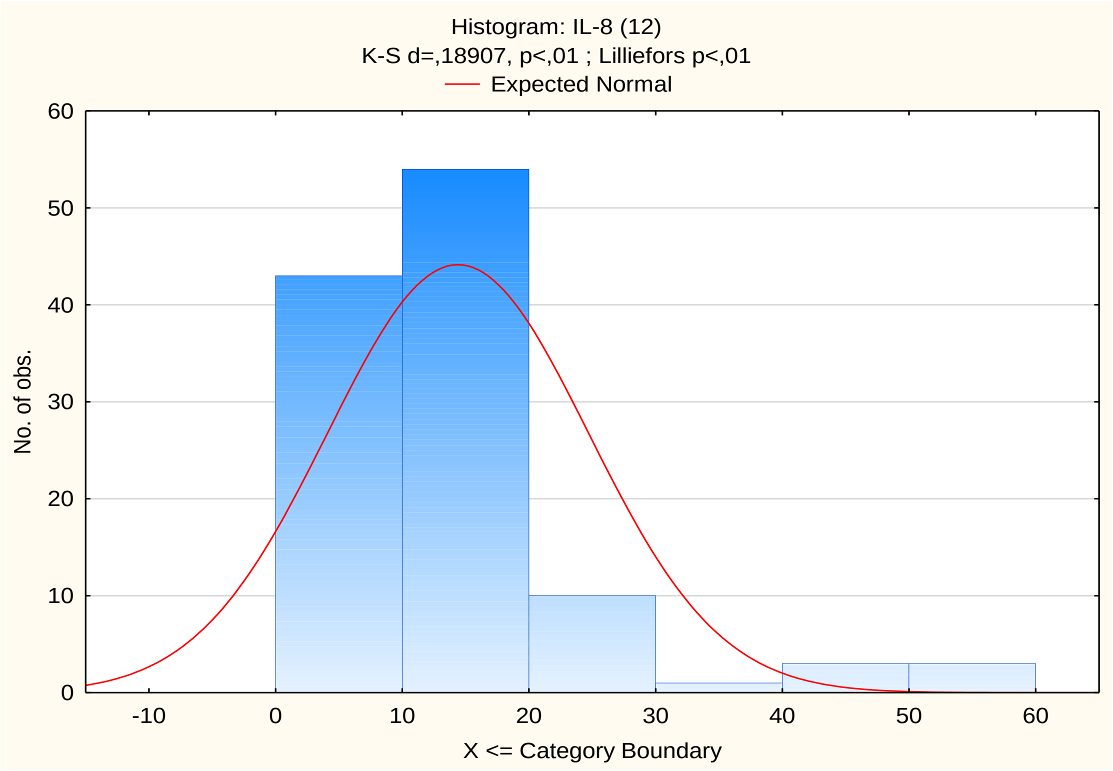

Figure S21. Normality plot of annual concentration of IL-8.

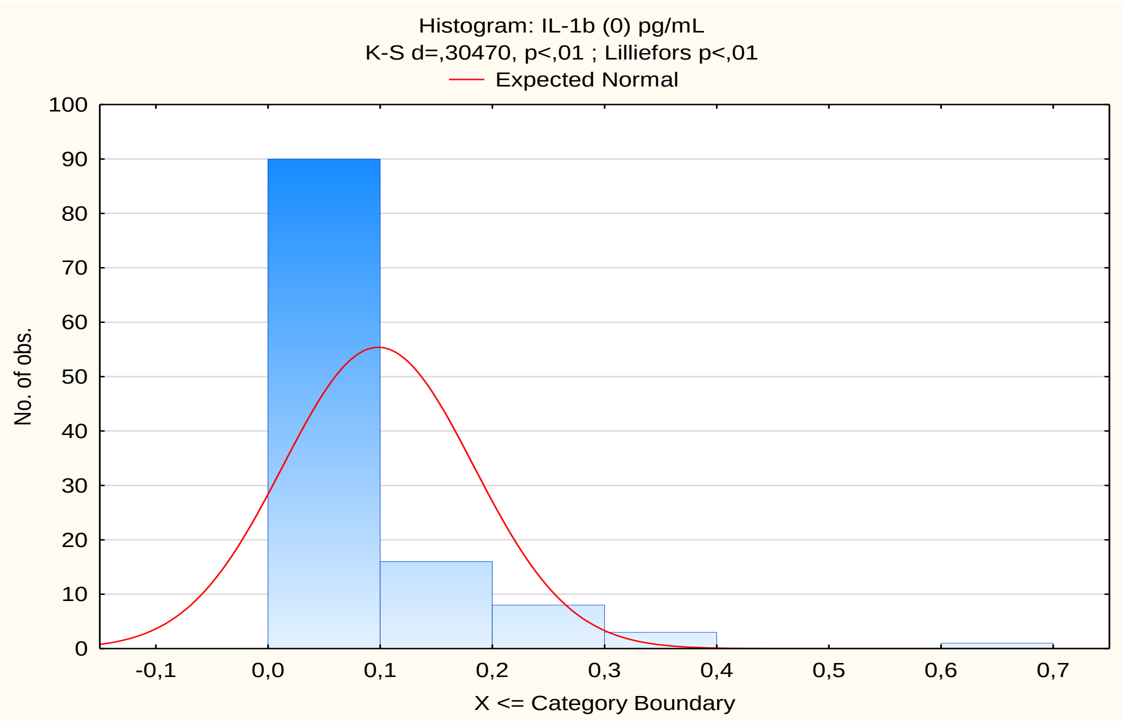

Figure S22. Normality plot of initial concentration of IL-1b.

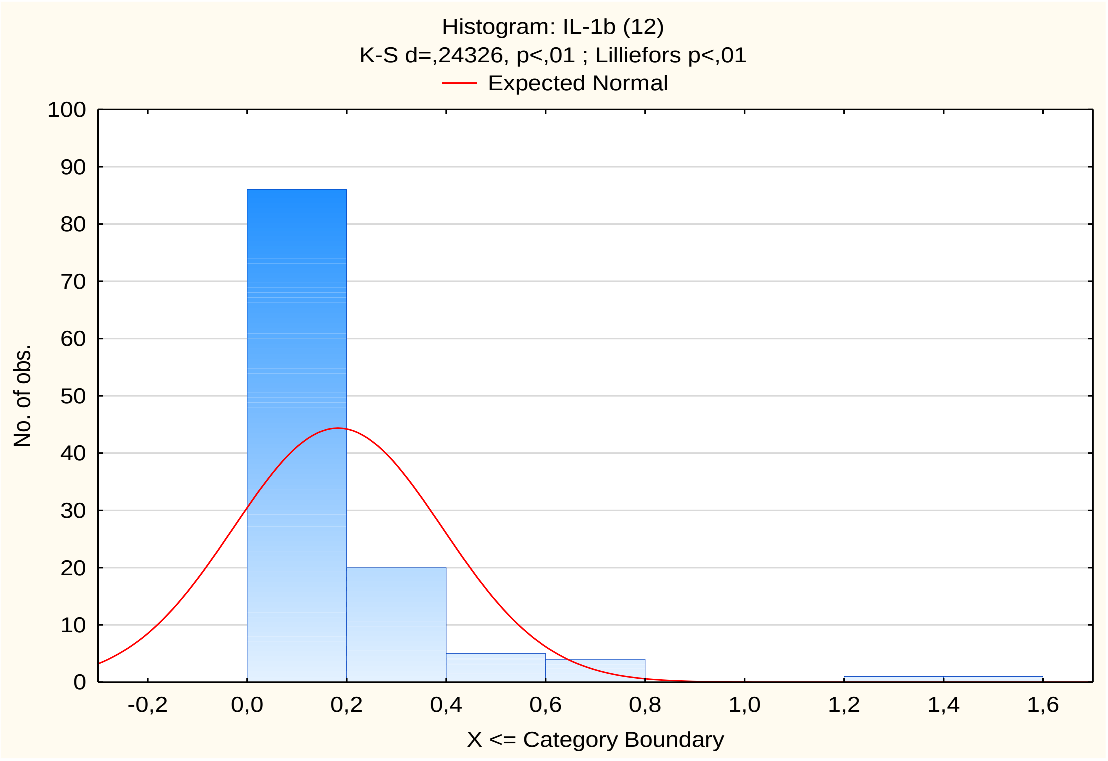

Figure S23. Normality plot of annual concentration of Il-1b.
