# Supplementary material for: The Role of Secreted Frizzled-Related Protein 5 (Sfrp5) in Overweight and Obesity in Childhood and Adolescence
Source: Nutrients. 2024 Sep 17;16(18):3133. doi: 10.3390/nu16183133 (PMC11434931; doi:10.3390/nu16183133)
Supplement: Supplementary file 1 [file nutrients-16-03133-s001.zip › nutrients-3131026_Supplemental tables.pdf]

SUPPLEMENTAL TABLES

Supplemental Table S1. Correlation coefficient analysis between assessed variables and Sfrp5 concentrations in the morbid obesity, obesity, overweight and normal BMI groups at initial assessment

Variables	Morbid Obesity	Obesity	Overweight	Normal BMI
BMI_t0	r=-0.06	**r=0.48 p<0.05**	r=0.08	r=0.45
SBP_t0	r=0.124522	**r=0.610178 p<0.05**	r=0.020031	r=0.127472
DBP_t0	r=0.127706	r=0.292513	r=0.154279	r=0.334533
Glucose_0	r=-0.109314	r=-0.195404	r=0.019002	r=-0.043940
AST_0	r=-0.044658	r=0.128164	r=-0.319106	r=0.223987
ALT_0	r=-0.028632	r=0.127385	r=-0.284451	r=0.074652
gGT_0	r=0.072656	r=0.179745	r=-0.073908	r=0.128060
ALP_0	r=-0.000954	r=-0.304184	r=0.137922	r=-0.418179
Albumin_0	r=0.171800	r=0.182613	r=-0.063030	r=-0.389307
Cholesterol_0	r=-0.138003	r=-0.005800	r=-0.261957	r=-0.351635
TG_0	r=-0.118365	r=0.352350	r=0.069002	**r=0.664710 p<0.05**
HDL_0	r=-0.119733	r=-0.288471	r=-0.029138	**r=-0.622783 p<0.05**
LDL_0	r=-0.024092	r=-0.049280	r=-0.255319	r=-0.013779
Uric_acid_0	r=0.175758	**r=0.706725 p<0.05**	r=-0.025232	r=0.237070
ApoA1_0	r=-0.105491	r=-0.318728	r=-0.053703	**r=-0.565480 p<0.05**
ApoB_0	r=-0.105778	r=-0.007640	r=-0.243057	r=0.008763
Lpa_0	r=-0.033183	r=0.314480	r=-0.125743	r=0.426238
Insulin_0	r=-0.051506	r=0.245419	r=0.357915	**r=0.610588 p<0.05**
HbA1c_0	r=-0.072851	r=0.202263	r=0.357406	r=0.087032
HOMA_0	r=-0.061247	r=0.205011	r=0.316586	**r=0.552901 p<0.05**
Hs-CRP (0)	r=-0.137369	r=-0.214635	r=-0.178912	r=-0.401512
TNF-α (0)	r=0.262957	r=0.366318	r=0.251430	**r=0.698965 p<0.05**
IL-6 (0)	r=-0.062761	r=0.204952	r=-0.127313	**r=0.568570 p<0.05**
TOS (0)	r=-0.021222	r=-0.229029	r=-0.190639	r=0.365020
TAS (0)	r=0.252425	r=0.166244	r=0.026466	r=-0.053019
IL-8 (0)	r=0.144331	r=0.378484	**r=0.488083 p<0.05**	r=0.484163
IL-1b (0)	r=0.131489	r=0.017304	r=-0.237796	r=0.487410
IL-1a (0)	r=0.008642	r=0.331382	r=-0.256363	NA
IL-2 (0)	r=-0.004584	r=0.220527	NA	NA
IL-12 (0)	**r=0.604027 p<0.05**	r=-0.169045	r=0.407061	r=-0.210354
Fat mass	r=-0.094955	r=0.238755	r=0.180129	**r=0.614121 p<0.05**

Abbreviations: ALP, alkaline phosphatase; ALT, alanine transaminase; Apo-A1, apolipoprotein A1; Apo-B, apolipoprotein B; AST, aspartate aminotransferase; BMI, Body Mass Index; DBP, diastolic blood pressure; hs-CRP, High sensitivity C-Reactive Protein; γGT, gamma-glutamyl transferase; HbA1C, hemoglobin A1C; HDL, high density lipoprotein; HOMA-IR, homeostatic model assessment for insulin resistance; IL, interleukin; LDL, low density lipoprotein; Lp(a), lipoprotein a; SBP, systolic blood pressure; SFRP5, Secreted Frizzled Related Protein 5; TAS, Total antioxidant status; TNF-α, tumor necrosis factor-a; TOS; Total oxidant status.

Correlations of the studied variables are evaluated by the Pearson's R coefficient, and presented by r. Statistical significance was set at $p < 0.05$; Statistically significant associations are shown in bold; NA, non-applicable

**Supplemental table S2.** Predictors of Sfrp5 and ΔSfrp5		
**Independent Variables**	**Dependent Variable (b)**	***p* Value**
Inflammation parameters at initial assessment (TAS, TOS, hs-CRP, TNF-α, IL-6)		
initial TNF-α<br>initial hs-CRP	initial Sfrp5 (b= 0.288)<br>initial Sfrp5 (b=-0.232)	$p < 0.05$
Inflammation parameters at annual assessment (TAS, TOS, hs-CRP, TNF-α, IL-6)		
annual TNF-α<br>annual TAS	annual Sfrp5 (b= 0.277)<br>annual Sfrp5 (b= 0.228)	$p < 0.05$
Metabolic syndrome parameters at initial assessment (glucose concentration, SBP, WC, TG, HDL)		
initial SBP	initial Sfrp5 (b= 0.233,	$p < 0.05$
When metabolic syndrome parameters at initial assessment (glucose concentration, SBP, WC, TG, HDL)		
initial glucose concentration	ΔSfrp5 (b= 0.208)	$p < 0.05$
